# SGLT2 inhibition alters substrate utilization and mitochondrial redox in healthy and failing rat hearts

**DOI:** 10.1172/JCI176708

**Published:** 2024-12-16

**Authors:** Leigh Goedeke, Yina Ma, Rafael C. Gaspar, Ali Nasiri, Jieun Lee, Dongyan Zhang, Katrine Douglas Galsgaard, Xiaoyue Hu, Jiasheng Zhang, Nicole Guerrera, Xiruo Li, Traci LaMoia, Brandon T. Hubbard, Sofie Haedersdal, Xiaohong Wu, John Stack, Sylvie Dufour, Gina Marie Butrico, Mario Kahn, Rachel J. Perry, Gary W. Cline, Lawrence H. Young, Gerald I. Shulman

**Affiliations:** 1 Department of Internal Medicine (Endocrinology), Yale School of Medicine, New Haven Connecticut, USA.; 2 Department of Medicine (Cardiology) and The Cardiovascular Research Institute and; 3 Department of Medicine (Endocrinology) and The Diabetes, Obesity and Metabolism Institute, Icahn School of Medicine at Mount Sinai, New York, New York, USA.; 4 Department of Internal Medicine (Cardiovascular Medicine) and The Yale Cardiovascular Research Center, Yale School of Medicine, New Haven Connecticut, USA.; 5 Department of Biomedical Sciences, Faculty of Health and Medical Sciences, University of Copenhagen, Copenhagen, Denmark.; 6 Department of Cellular & Molecular Physiology, Yale School of Medicine, New Haven Connecticut, USA.; 7 Department of Clinical Research, Copenhagen University Hospital, Steno Diabetes Center Copenhagen, Herlev, Denmark.; 8 Howard Hughes Medical Institute, Chevy Chase, Maryland, USA

**Keywords:** Cardiology, Metabolism, Glucose metabolism, Intermediary metabolism, Mitochondria

## Abstract

Previous studies highlight the potential for sodium-glucose cotransporter type 2 (SGLT2) inhibitors (SGLT2i) to exert cardioprotective effects in heart failure by increasing plasma ketones and shifting myocardial fuel utilization toward ketone oxidation. However, SGLT2i have multiple in vivo effects and the differential impact of SGLT2i treatment and ketone supplementation on cardiac metabolism remains unclear. Here, using gas chromatography–mass spectrometry (GC-MS) and liquid chromatography–tandem mass spectrometry (LC-MS/MS) methodology combined with infusions of [^13^C_6_]glucose or [^13^C_4_]βOHB, we demonstrate that acute SGLT2 inhibition with dapagliflozin shifts relative rates of myocardial mitochondrial metabolism toward ketone oxidation, decreasing pyruvate oxidation with little effect on fatty acid oxidation in awake rats. Shifts in myocardial ketone oxidation persisted when plasma glucose levels were maintained. In contrast, acute βOHB infusion similarly augmented ketone oxidation, but markedly reduced fatty acid oxidation and did not alter glucose uptake or pyruvate oxidation. After inducing heart failure, dapagliflozin increased relative rates of ketone and fatty acid oxidation, but decreased pyruvate oxidation. Dapagliflozin increased mitochondrial redox and reduced myocardial oxidative stress in heart failure, which was associated with improvements in left ventricular ejection fraction after 3 weeks of treatment. Thus, SGLT2i have pleiotropic effects on systemic and heart metabolism, which are distinct from ketone supplementation and may contribute to the long-term cardioprotective benefits of SGLT2i.

## Abstract 2

This study reveals that SGLT2 inhibitors and ketone infusions affect heart metabolism differently, with SGLT2 inhibition having both acute and lasting effects in heart failure.

## Graphical abstract

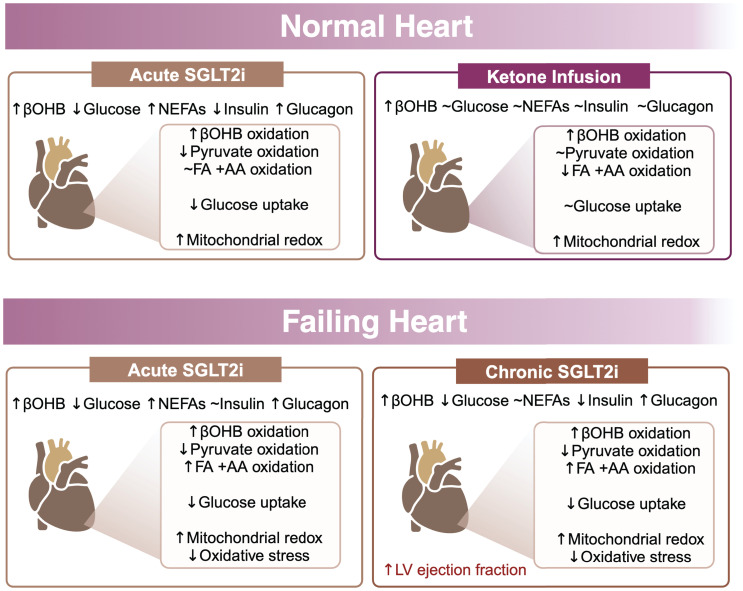

## Introduction

Sodium-glucose cotransporter type 2 (SGLT2) inhibitors (SGLT2i) block glucose reabsorption in the renal proximal tubule, thereby inducing glycosuria and natriuresis and leading to reductions in hyperglycemia, body weight, and blood pressure (1). More recently, large clinical trials have demonstrated that SGLT2i have beneficial effects on cardiovascular outcomes in patients with both reduced and preserved ejection fraction when added to standard-of-care therapy (2–8). However, the mechanisms responsible for the beneficial effects of SGLT2i on heart failure remain debated.

One proposed mechanism is that SGLT2 inhibition shifts cardiomyocyte energy metabolism toward ketone utilization with potential improvement in bioenergetic capacity and cardiac efficiency in the failing heart (9–11). The myocardium requires large amounts of energy derived from nutrient metabolism to fuel cardiac contraction and maintain transmembrane ionic gradients (12). To generate adequate amounts of adenosine triphosphate (ATP), the normal heart is extremely flexible and can adapt to altered fuel supply and hormonal concentrations (13). Cardiac mitochondria are capable of oxidizing fatty acids, pyruvate (derived from glucose and/or lactate), ketone bodies, and amino acids depending on energy demand, substrate availability, and/or circulating hormone concentrations (14). Although fatty acids are considered to be the predominant fuel source for normal adult hearts in the fasting state, several physiologic and pathologic conditions increase the contribution of other substrates for ATP production (13). In heart failure, the balance of myocardial fatty acid oxidation, pyruvate oxidation, and glycolysis is altered, depending on the duration and severity of disease (13). More recently, enhanced myocardial ketone oxidation has been shown to be an adaptive response in both experimental and clinical heart failure (15–19). In light of these studies, ketones have been hypothesized to be a “thrifty-fuel” (9), with increases in ketone availability (either directly by ketone infusion or indirectly through SGLT2 inhibition) being demonstrated to reduce pathologic cardiac remodeling and improve systolic function in murine, canine, and swine models of heart failure (15, 20, 21). In addition, acute ketone supplementation has also been shown to increase cardiac output in humans with heart failure (22).

Most prior studies investigating cardiac metabolism in heart failure have not measured myocardial substrate oxidation rates in the intact heart in awake animals. Instead, they have relied on ex vivo measurements of substrate oxidation using isolated cardiomyocytes or working heart perfusions (23–25) or static measurements of metabolites and/or changes in metabolic enzymes (16, 26–29). Of note, in vitro studies do not replicate the changes in circulating hormones and substrates that occur in heart failure and have a critical impact on cardiac metabolism. Thus, a fully integrated in vivo approach is needed to understand the overall impact of substrate delivery, hormonal alterations, and cardiac metabolic remodeling on heart metabolism, along with the impact of SGLT2i treatment. The measurement of differences in blood arterial and coronary venous metabolite concentrations provides an overall assessment of substrate extraction or production in vivo, but sampling of coronary venous blood is not feasible in rodents, limiting the application of this technique to small animal models (21). Moreover, in patients, the use of anesthesia confounds metabolic substrate metabolism (17, 21). Recent in vivo stable isotope tracer studies tracking cardiac glucose and branched chain amino metabolism in conscious rodents have circumvented some of these methodological limitations (29–34), but to date, the determinants of in vivo cardiac βOHB utilization in the awake, unrestrained state have not been determined, limiting the assessment of ketone metabolism during SGLT2 inhibition.

Thus, using gas chromatography-mass spectrometry (GC-MS) and liquid chromatography–tandem mass spectrometry (LC-MS/MS) methodology combined with infusions of [^13^C_6_]glucose or [^13^C_4_]βOHB, we developed and utilized a method to assess the relative rates of myocardial mitochondrial pyruvate (derived from glucose and lactate) and ketone oxidation in awake rats. We compared the relative utilization of myocardial pyruvate, ketones, and fatty acids both in healthy rats and in rats following the induction of heart failure after myocardial infarction (MI). We then used this method to elucidate the acute effects of SGLT2 inhibition on myocardial mitochondrial substrate oxidation in both healthy and heart-failure rats. We tested the hypothesis that SGLT2i-mediated increases in hepatic ketogenesis would lead to increased βOHB availability and oxidation and evaluated whether the myocardial fuel switch to ketone bodies was accompanied by a decrease in pyruvate (derived from glucose and lactate) and/or fatty acid oxidation. The contribution of glucose lowering to the effects of SGLT2i on substrate oxidation was further investigated by concomitant glucose infusion to maintain euglycemia. The effects of SGLT2i treatment were then compared with those of acute ketone infusions to achieve comparable plasma ketone levels, in order to better understand how additional metabolic changes might contribute to the SGLT2i effects on heart metabolism. Finally, we assessed the extent to which the shift in myocardial substrate utilization by SGLT2i was sustained with chronic therapy and whether SGLT2i would acutely or chronically improve cardiac contractile function in the setting of heart failure.

## Results

### Measurement of relative rates of cardiac-specific substrate oxidation in vivo.

We first developed a combined stable isotope tracer and mass spectrometry approach to assess relative rates of myocardial substrate utilization in chow-fed awake male Sprague-Dawley rats, based on previously established methodology in skeletal muscle (35–37). Following a 6-hour fast, a steady-state infusion of [^13^C_6_]glucose was initiated and continued for 2 hours; hearts were excised and the myocardial ^13^C enrichments of glutamate and alanine were assessed for the calculation of mitochondrial pyruvate dehydrogenase flux (V_PDH_) to total mitochondrial oxidation (V_CS_, citrate synthase flux) (Supplemental Figure 1, A–G; supplemental material available online with this article; https://doi.org/10.1172/JCI176708DS1). Metabolism of [^13^C_6_]glucose produces [^13^C_3_]pyruvate (m+3) via glycolysis that enters the TCA cycle via pyruvate dehydrogenase (PDH) to produce [^13^C_2_]acetyl-CoA (m+2). The ratio of [^13^C_2_]acetyl-CoA (m+2) to [^13^C_3_]pyruvate (m+3) reflects carbohydrate flux through V_PDH_ relative to V_CS_ (V_PDH_/V_CS_), such that a ratio of one indicates 100% pyruvate oxidation (derived from glucose/lactate) and any ratio less than one reflects dilution of the tracer from unlabeled acetyl-CoA derived from the oxidation of fatty acids, ketones, and/or ketogenic amino acids (Supplemental Figure 1A). Since pyruvate and acetyl-CoA enrichments are small and difficult to reliably measure in tissues, we assessed the enrichment of their reciprocal pools, [^13^C_3_]alanine (m+3) and [4,5-^13^C_2_]glutamate (m+2) (35, 36), for an estimation of myocardial V_PDH_/V_CS_ (Supplemental Figure 1G). Importantly, in separate experiments, we found that [^13^C_3_]alanine equilibrated with [^13^C_3_]pyruvate (m+3) (Supplemental Figure 1C) while [4,5-^13^C_2_]glutamate (m+2), equilibrated with [^13^C_2_]acetyl-CoA (m+2) (Supplemental Figure 1D), validating the use of reciprocal [^13^C]metabolite pools for our measurements (35, 36).

Using a similar approach, we next assessed the relative contribution of ketone oxidation (V_BDH_) to total mitochondrial oxidation (V_CS_) following a 2-hour steady-state infusion of [^13^C_4_]βOHB in a separate cohort of rats (Supplemental Figure 1, H–N). In the heart, β-hydroxybutyrate dehydrogenase (BDH1) catalyzes the oxidation of [^13^C_4_]βOHB to [^13^C_4_]acetoacetate (m+4), which is subsequently transferred onto CoA through the activity of succinyl-CoA:3-oxoacid-CoA transferase (SCOT) and catalyzed by mitochondrial thiolase into 2 molecules of [^13^C_2_]acetyl-CoA (m+2). The ratio of [^13^C_2_]acetyl-CoA (m+2) to [^13^C_4_]βOHB (m+4) reflects ketone flux of βOHB (V_BDH_) relative to V_CS_ (V_BDH_ /V_CS_), such that a ratio of one indicates 100% ketone oxidation and any ratio less than one reflects dilution of the tracer from unlabeled acetyl-CoA derived from the oxidation of other substrates such as glucose, pyruvate, lactate, fatty acids, and ketogenic amino acids (Supplemental Figure 1H). While myocardial [^13^C_4_]βOHB (m+4) enrichments can reliably be measured following a steady-state infusion of [^13^C_4_]βOHB (Supplemental Figure 1, J and N), [4,5-^13^C_2_]glutamate (m+2) was again used as a surrogate for [^13^C_2_]acetyl-CoA (m+2) (Supplemental Figure 1, K and N).

Based on these measurements, we next calculated the relative rates of cardiac mitochondrial substrate oxidation. As shown in Supplemental Figure 1, O–Q, relative rates of V_PDH_/V_CS_ were 45%, while relative rates of V_BDH_ /V_CS_ were 13%. The relative oxidation rate of fatty acids and ketogenic amino acids (V_FFA_ _+AA_) to total mitochondrial oxidation (V_CS_) was indirectly assessed by calculating the residual V_CS_ that was not accounted for after combining the myocardial V_BDH_/V_CS_ and V_PDH_/V_CS_ obtained during the [^13^C_6_]glucose and [^13^C_4_]βOHB infusions, i.e., [1- (V_BDH_ /V_CS_ + V_PDH_ /V_CS_)]. Using this approach, we estimated that fatty acid and ketogenic amino acid oxidation (V_FFA_ _+AA_) accounts for 42% of total cardiac mitochondrial oxidation in chow-fed rats (Supplemental Figure 1Q). Together, these results indicate that during brief fasting (6–8 hours), a healthy rodent heart oxidizes 3-to-4-fold more glucose and lactate-derived pyruvate (~45%) compared with ketones (~13%) in the awake state, while also indicating less reliance on the oxidation of fatty acids than hearts perfused in vitro with free fatty acids and glucose (38). These relative oxidation rates are in accordance with early studies of human cardiac substrate metabolism in vivo (39–42).

### Acute dapagliflozin treatment increases plasma ketone availability and myocardial ketone oxidation.

**Figure 1:**
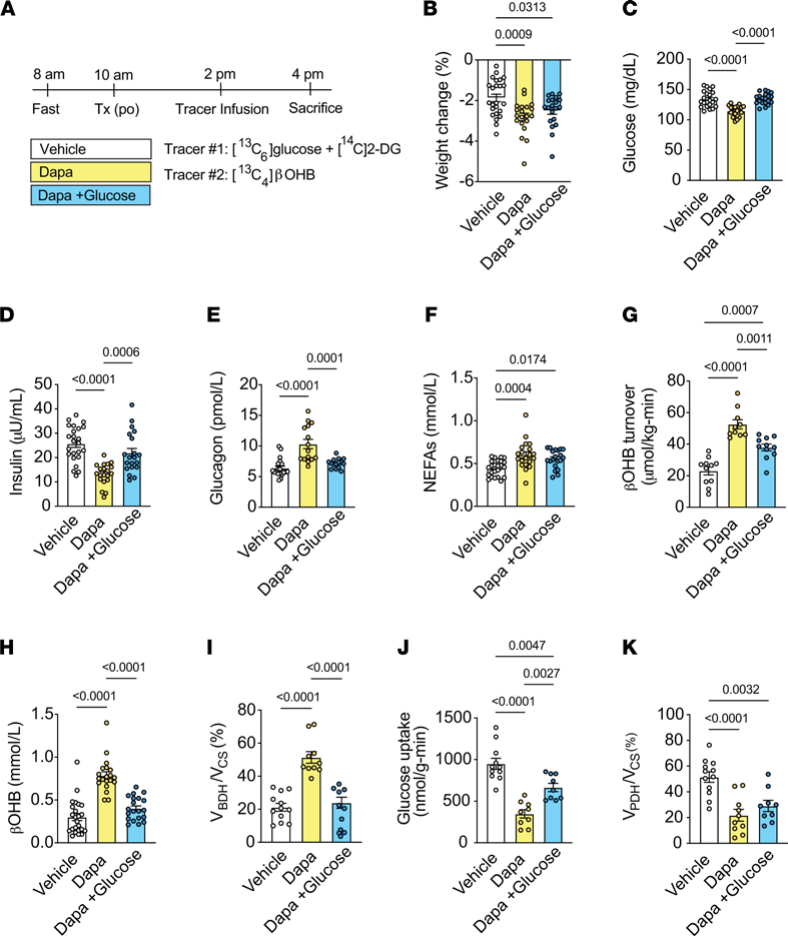
Acute dapagliflozin treatment significantly increases relative rates of β**OHB oxidation at the expense of pyruvate oxidation in chow-fed male rats.** (**A**) Outline of study design. Rats were given an intravenous bolus of ^14^C[2-DG] during the last 20 minutes of the infusion. (**B**–**K**) Weight change (**B**), plasma glucose (**C**), plasma insulin (**D**), plasma glucagon (**E**), plasma NEFAs (**F**), whole-body βOHB turnover (**G**), plasma βOHB (**H**), relative rates of myocardial βOHB oxidation (V_BDH_) to total mitochondrial oxidation (V_CS_) (**I**), myocardial glucose uptake (**J**), and relative rates of myocardial pyruvate oxidation (V_PDH_) to total mitochondrial oxidation (V_CS_) (**K**) in chow-fed male rats treated as in **A**. In panels (**B**–**K**), *n* = 24, 20, 20 (**B** and **D** and **F**); *n* = 22, 20, 20 (**C**); *n* = 24, 19, 19 (**E**); *n* = 11, 10, 11 (**G**); *n* = 22, 19, 20 (**H**); *n* = 12, 10, 11 (**I**); *n* = 11, 9, 9 (**J**); and *n* = 12, 10, 9 (**K**). All data are represented as mean ± SEM. *P* < 0.05 by 1-way ANOVA with Bonferroni’s corrections for multiple comparisons. Dapa, dapagliflozin; Tx, treatment; po, orally.

**Figure 2:**
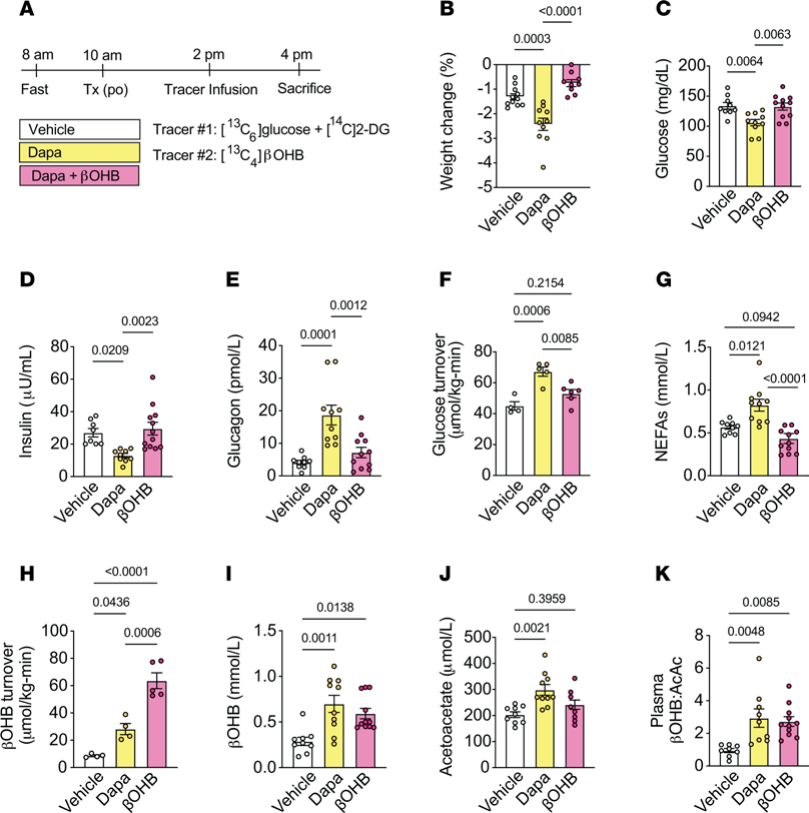
βOHB infusion significantly increases relative rates of βOHB oxidation in chow-fed male rats. (**A**) Outline of study design. Rats were given an intravenous bolus of ^14^C[2-DG] during the last 20 minutes of the infusion. (**B**–**K**) Weight change (**B**), plasma glucose (**C**), plasma insulin (**D**), plasma glucagon (**E**), whole-body glucose turnover (**F**), plasma NEFAs (**G**), whole-body βOHB turnover (**H**), plasma βOHB (**I**), plasma acetoacetate (**J**), and plasma βOHB:AcAc in chow-fed male rats treated as in **A**. In panels (**B**–**K**), *n* = 11, 10, 9 (**B**); *n* = 9, 10, 13 (**C**); *n* = 8, 9, 12 (**D**); *n* = 9, 10, 11 (**E**, **G**, and **I**); *n* = 4, 5, 6 (**F**); *n* = 4, 4, 5 (**H**); *n* = 9, 10, 10 (**J**); and *n* = 8, 9, 11 (**K**). All data are represented as mean ± SEM. *P* < 0.05 by 1-way ANOVA with Bonferroni’s corrections for multiple comparisons.

To examine the effect of acute SGLT2 inhibition on in vivo cardiac mitochondrial metabolism, we treated 2-hour–fasted awake chow-fed rats with a single oral dose of 1.5 mg/kg dapagliflozin, a dose that achieved clinically relevant plasma concentrations of dapagliflozin (~0.8 μM [Supplemental Figure 1, R–S] versus ~0.4 μM in humans) (43). The rats were infused 4 hours later with [^13^C_6_]glucose (for an additional 2 hours) and given a bolus of [^14^C]2-deoxy-d-glucose (2-DG) during the last 20 minutes of the infusion to assess relative rates of myocardial V_PDH_/V_CS_ and glucose uptake, respectively (Figure 1A). A separate group of rats were infused with [^13^C_4_]βOHB to assess relative rates of myocardial V_BDH_/V_CS_ and whole-body βOHB turnover (Figure 2A). Six hours after treatment, there was marked accumulation of dapagliflozin in the kidney and a consequent 3- to 4-fold increase in plasma βOHB levels (Supplemental Figure 1, S and T). Consistent with the known metabolic effects of SGLT2i, acute dapagliflozin treatment led to reductions in fasting plasma glucose and insulin concentrations and a small but detectable weight loss due to glycosuria (Figure 1, B–D, and Supplemental Figure 2A). Dapagliflozin treatment also reduced the insulin/glucagon ratio and increased whole-body glucose turnover compared with what occurred in vehicle-treated control rats (Figure 1, D and E, and Supplemental Figure 2, B and C). These parameters were associated with an increase in fasting plasma nonesterified fatty acid (NEFA) concentrations and hepatic ketogenesis, as evidenced by a marked increase in whole-body βOHB turnover, plasma βOHB concentrations, and plasma acetoacetate levels in dapagliflozin-treated rats compared with controls (Figure 1, F–H, and Supplemental Figure 2D). βOHB clearance was not significantly altered between treatment groups (Supplemental Figure 2E). Notably, dapagliflozin treatment increased V_BDH_/V_CS_ by 2-fold from approximately 20% to approximately 51% (Figure 1I). Acute dapagliflozin treatment also reduced myocardial glucose uptake, as assessed by [^14^C]2-DG accumulation, and V_PDH_/V_CS_ by 60% compared with vehicle controls (Figure 1, J and K). In contrast, relative rates of V_FFA_ _+AA_/V_CS_ remained unchanged (Supplemental Figure 2H). These results indicate that in vivo dapagliflozin treatment augments myocardial oxidation of ketones relative to pyruvate (derived from glucose/lactate) rather than by diminishing relative fatty acid oxidation.

### Dapagliflozin-mediated reductions in relative rates of cardiac pyruvate oxidation are independent of plasma glucose concentrations.

Acute dapagliflozin-mediated reductions in glucose uptake and relative rates of V_PDH_/V_CS_ could be driven by reductions in plasma glucose/insulin and/or increases in plasma ketone availability and myocardial ketone oxidation. In order to differentiate between these 2 possibilities, a separate group of dapagliflozin-treated rats was also given a variable infusion of glucose, which matched plasma glucose and insulin concentrations to those of vehicle-treated rats (~125 mg/dL and 20 μU/ml, respectively; herein referred to as dapagliflozin + glucose) (Figure 1, A, C, and D).

The exogenous infusion of glucose reduced dapagliflozin-mediated increases in plasma glucagon, whole-body βOHB turnover, plasma βOHB concentrations, and plasma acetoacetate concentrations, independently of changes in βOHB clearance (Figure 1, E–H, and Supplemental Figure 2, B–E). Interestingly, plasma NEFA levels and whole-body glucose turnover remained increased (Figure 1F and Supplemental Figure 2C), most likely due to only a partial restoration of the insulin/glucagon ratio in dapagliflozin-treated rats infused with glucose compared with vehicle controls (Supplemental Figure 2B). Although dapagliflozin + glucose also increased myocardial glucose uptake, it remained partially suppressed compared with vehicle-treated rats (Figure 1J), and dapagliflozin + glucose had no significant effect to increase myocardial V_PDH_/V_CS_, which remained lower than in vehicle controls (Figure 1K). Relative rates of V_FFA+AA_/V_CS_ increased in dapagliflozin + glucose–treated versus vehicle-treated control rats and dapagliflozin-treated rats (~47% versus 31% and 23%; Supplemental Figure 2H) despite the plasma glucose and insulin levels being higher than in dapagliflozin-treated rats and similar to that in vehicle-treated rats (Figure 2D). In contrast to the heart, exogenous glucose infusion significantly increased glucose uptake in white adipose tissue (eWAT) and skeletal muscle in rats treated with dapagliflozin (Supplemental Figure 2, F and G). The differential effect of glucose infusion to suppress glucose uptake in heart as compared with adipose or skeletal muscle likely reflects the more prominentRandle effect (44) of residually elevated plasma NEFA concentrations in the heart (Figure 1F).

In contrast, the elevated relative rate of myocardial βOHB oxidation in dapagliflozin-treated rats was reduced by exogenous glucose infusion, which partially normalized hepatic ketogenesis and fasting plasma βOHB levels to those in vehicle-treated rats (Figure 1, G and H). Collectively, these data suggest that dapagliflozin-mediated increases in cardiac βOHB oxidation are dependent on plasma glucose/insulin concentrations and presumably are driven primarily by increases in plasma ketone availability resulting from increased hepatic ketogenesis. They also suggest that the relative suppression of myocardial pyruvate oxidation during dapagliflozin treatment might be related to competition from elevated plasma NEFA levels rather than reduced plasma glucose and insulin levels. In addition, reductions in myocardial pyruvate oxidation may be driven by competition from increased circulating ketones, which were only partially corrected with exogenous glucose infusion (Figure 1H and Supplemental Figure 2D).

### Contribution of elevated plasma ketones to the effects of dapagliflozin on cardiac metabolism.

**Figure 3:**
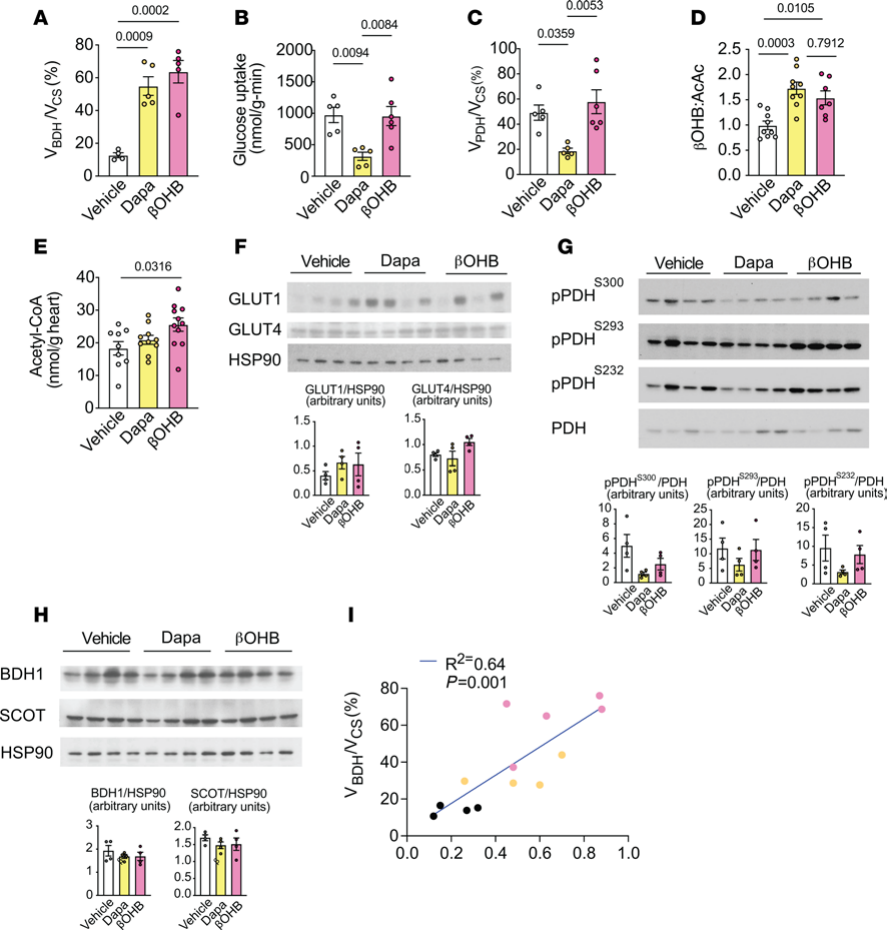
Acute dapagliflozin-mediated increases in relative rates of β**OHB oxidation are driven by plasma** β**OHB levels.** (**A**–**E**) Relative rates of myocardial βOHB oxidation (V_BDH_) to total mitochondrial oxidation (V_CS_) (**A**), myocardial glucose uptake (**B**), relative rates of myocardial pyruvate oxidation (V_PDH_) to total mitochondrial oxidation (V_CS_) (**C**), myocardial βOHB:AcAc (**D**), and myocardial acetyl-CoA content (**E**) in chow-fed male rats treated with vehicle, 1.5 mg/kg body weight dapagliflozin (po), or infused with 50 μmol/(kg-min) βOHB. (**F**–**H**) Representative Western blot analysis of GLUT1 and GLUT4 (**F**), pPDH/PDH (**G**), and BDH1 and SCOT (**H**) in the hearts of chow-fed male rats treated as in **A**–**E**. HSP90 was used as a loading control. Quantification of blots shown below each blot. (**I**) Correlation of plasma βOHB and V_BDH_/ V_CS_ in rats treated as in **A**–**E**. In panels (**A**–**I**), *n* = 4, 4, 5 (**A**); *n* = 5, 5, 6 (**B** and **C**); *n* = 9, 9, 7 (**D**); *n* = 9, 12, 13 (**E**); *n* = 4 per group (**F**–**H**); and *n* = 4, 4, 5 (**I**). All data are represented as mean ± SEM. *P* < 0.05 y 1-way ANOVA with Bonferroni’s corrections for multiple comparisons.

To further assess the impact of elevated circulating ketones per se in driving alterations in cardiac substrate oxidation in vivo, we next assessed mitochondrial substrate oxidation in a separate cohort of rats treated with vehicle or dapagliflozin or infused with 50 μmol/kg-min exogenous βOHB to match βOHB levels to those measured in dapagliflozin-treated rats (~0.6 mM) (Figure 2, A and I). In contrast to dapagliflozin treatment, exogenous βOHB infusion had no effect on body weight, fasting plasma glucose, plasma insulin, plasma glucagon, glucose turnover, or plasma NEFA levels when compared with vehicle controls (Figure 2, B–G, and Supplemental Figure 3A). As shown in Figure 2, H–J, and Supplemental Figure 3B, whole-body βOHB turnover and plasma βOHB levels were increased without evident changes in plasma or myocardial acetoacetate levels. βOHB infusion caused a 2-fold increase in myocardial βOHB concentrations and markedly increased V_BDH_/V_CS_ from 13% to 63% (Figure 3A and Supplemental Figure 3C). In contrast to dapagliflozin, which increased ketone oxidation at the expense of glucose uptake and relative pyruvate oxidation (Figure 1, J and K), exogenous βOHB infusion did not significantly decrease myocardial glucose uptake or V_PDH_/V_CS_ (Figure 3, B and C). However, in the absence of an increase in plasma NEFA levels that was seen with dapagliflozin, βOHB infusion substantially reduced fatty acid and ketogenic amino acid oxidation (Supplemental Figure 3D).

Plasma and myocardial mitochondrial redox (NADH:NAD^+^, as assessed by βOHB:AcAc) (45, 46), were also increased with βOHB infusion (Figure 2K and Figure 3D), consistent with increased extracellular βOHB leading to modulation of the intracellular mitochondrial redox state (47–49). βOHB infusion also increased myocardial acetyl-CoA content and oxidation of myocardial βOHB (Figure 3, A and E). This increase in myocardial βOHB oxidation during acute βOHB infusion was observed without evident changes in BDH1 and SCOT protein expression (Figure 3H). In contrast, protein expression of the cardiac glucose transporters GLUT1 and GLUT4 tended to be numerically higher (Figure 3F), consistent with previous studies in isolated human and mouse cardiomyocytes (50). The inhibitory phosphorylation of pyruvate dehydrogenase (PDH), the rate limiting enzyme for pyruvate oxidation, also trended down with βOHB infusion and acute dapagliflozin treatment (Figure 3G); however, neither trend was directly reflected in vivo by an increase in myocardial glucose uptake or the relative rate of pyruvate oxidation. Myocardial cytosolic redox (lactate:pyruvate) (Supplemental Figure 3, E–G), short chain CoA esters (Supplemental Figure 3H), anaplerosis-dependent substrates (Supplemental Figure 3, I–K), and ATP:ADP/ATP:AMP ratios (Supplemental Figure 3, L and M) could also not account for changes in cardiac substrate utilization associated with acute increases in βOHB availability. Collectively, these studies indicate that dapagliflozin-mediated increases in relative rates of myocardial ketone utilization are driven mainly by plasma ketone availability resulting from enhanced hepatic ketogenesis. Consistent with this conclusion, circulating plasma concentrations of βOHB positively correlated with the relative rates of myocardial βOHB oxidation (Figure 3I).

### Acute dapagliflozin treatment increases plasma ketone availability and myocardial ketone oxidation during heart failure.

**Figure 4:**
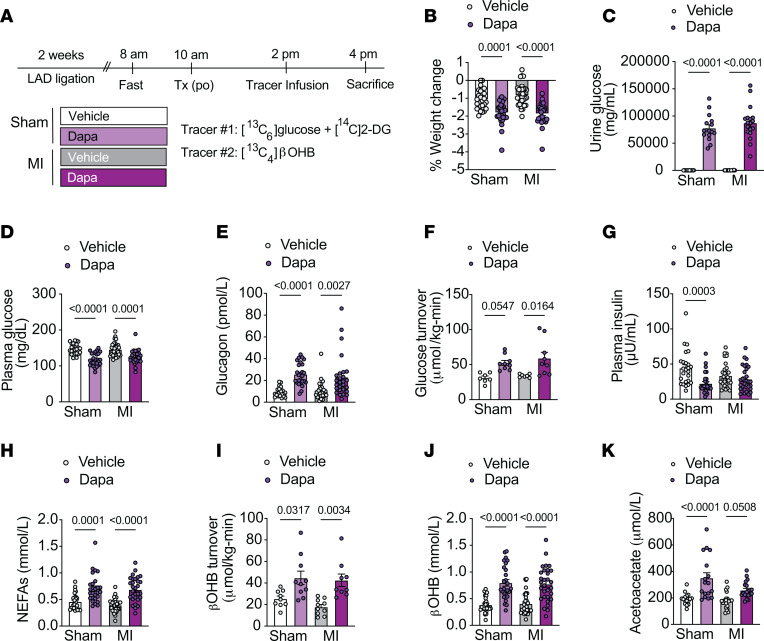
Acute dapagliflozin treatment increases hepatic ketogenesis and plasma ketone levels in sham-surgery and MI rats. (**A**) Outline of study design. Rats were given an intravenous bolus of ^14^C[2-DG] during the last 20 minutes of the infusion. (**B**–**K**) Weight change (**B**), urine glucose (**C**), plasma glucose (**D**), plasma glucagon (**E**), whole-body glucose turnover (**F**), plasma insulin (**G**), plasma NEFAs (**H**), whole-body βOHB turnover (**I**), plasma βOHB (**J**), and plasma acetoacetate (**K**) in chow-fed male rats treated as in **A**. In panels **B**–**K**, *n* = 26, 27, 33, 29 (**B**); *n* = 18, 16, 21,18 (**C**); *n* = 24, 26, 32, 29 (**D**); *n* = 22, 26, 28, 27 (**E**); *n* = 7, 9, 7, 9 (**F**); *n* = 25, 27, 32, 30 (**G**); *n* = 26, 27, 33, 30 (**H** and **J**); *n* = 9, 10, 10, 9 (**I**);and *n* = 16, 17, 21, 19 (**K**). All data are represented as mean ± SEM. *P* < 0.05 by 1-way ANOVA with Bonferroni’s corrections for multiple comparisons.

Having demonstrated that dapagliflozin shifts myocardial substrate utilization toward ketones in normal awake chow-fed rats, we next sought to directly assess its effects on cardiac mitochondrial substrate oxidation in heart failure. To this end, we utilized a well-characterized model of left anterior descending (LAD) coronary artery ligation, leading to MI and subsequent heart failure (51). Heart failure was evidenced 2 weeks after MI by significant reductions in left ventricular (LV) ejection fraction, fractional shortening, cardiac output, and stroke volume compared with sham-surgery controls (Supplemental Table 1). LV end-diastolic and end-systolic volumes were also markedly increased in heart-failure rats compared with sham-surgery rats, indicative of pathological cardiac remodeling (Supplemental Table 1). Sham-surgery controls and rats with heart failure (LV ejection fraction 72% ± 1% versus 41% ± 1%, respectively) were randomized to acute treatment with dapagliflozin or vehicle. For metabolic assessment, rats were infused with [^13^C_6_] glucose and given a bolus of [^14^C]2-DG to assess V_PDH_/V_CS_ and myocardial glucose uptake, respectively, or alternatively infused with [^13^C_4_]βOHB to assess V_BDH_/V_CS_ (Figure 4A). Similarly to what occurred in normal chow-fed rats, acute dapagliflozin treatment induced glycosuria, decreased plasma glucose, and reduced body weight in both sham-surgery and heart-failure rats compared with vehicle-treated rats (Figure 4, B–D, and Supplemental Figure 4A). Acute dapagliflozin treatment also increased plasma glucagon concentrations, lowered the insulin-to-glucagon ratio, and increased whole-body glucose turnover in sham-surgery and heart-failure rats (Figure 4, E and F, and Supplemental Figure 4B). However, significant reductions in plasma insulin concentrations were only observed in sham-surgery rats (Figure 4G). Dapagliflozin treatment caused a 2-fold increase in fasting plasma NEFA concentrations and whole-body βOHB turnover and increases in plasma βOHB and acetoacetate concentrations in the absence of changes in ketone clearance in both groups (Figure 4, H–K, and Supplemental Figure 4C).

**Figure 5:**
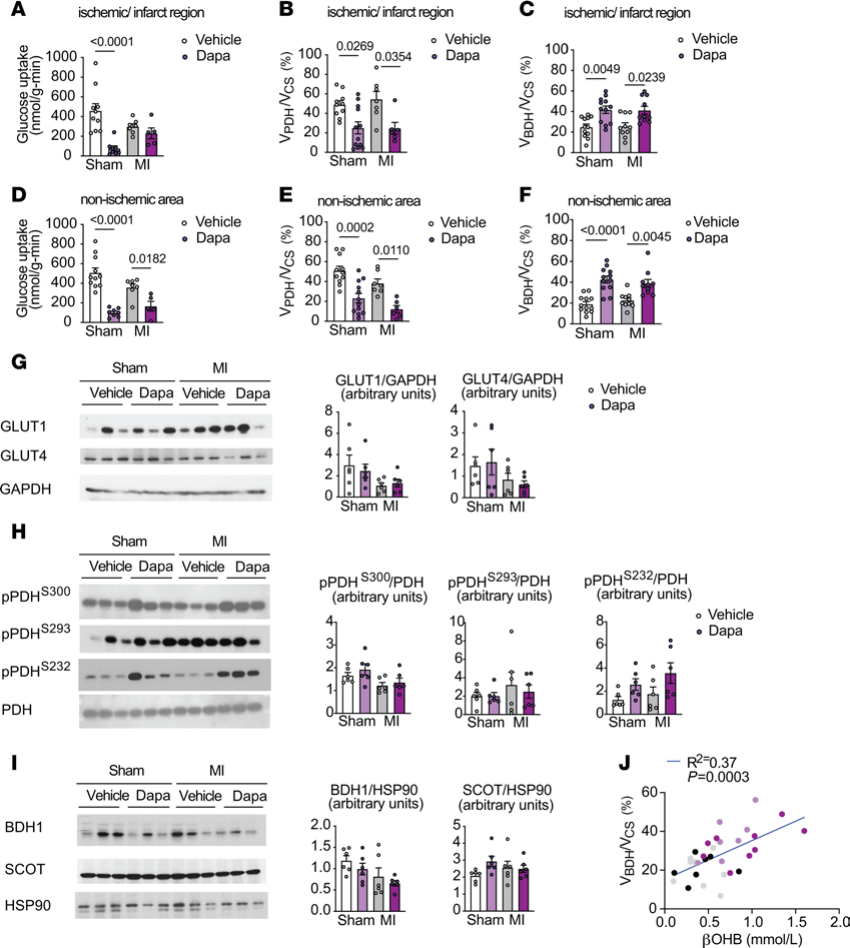
Acute dapagliflozin treatment reduces and increases relative rates of glucose and βOHB oxidation, respectively, in the ischemic/infarct area and nonischemic myocardium remote from the infarct area 2 weeks after MI. (**A**–**F**) Glucose uptake, relative rates of myocardial pyruvate oxidation (V_PDH_) to total mitochondrial oxidation (V_CS_), and relative rates of βOHB oxidation (V_BDH_) to total mitochondrial oxidation (V_CS_) in the ischemic/infarct region (**A**–**C**) and nonischemic myocardium remote from the infarct area (**D**–**F**) in chow-fed male rats 2 weeks after MI or sham surgery. (**G**–**I**) Representative Western blot analysis of GLUT1 and GLUT4 (**G**), pPDH/PDH (**H**), and BDH1 and SCOT (**I**) in the LV of sham and MI rats treated as in **A**–**F**. HSP90 was used as a loading control. Quantification of blots shown in the right panels. (**J**) Correlation of plasma βOHB and V_BDH_/V_CS_ in rats treated as in **A**–**F**. In panels **A**–**J**, *n* = 10, 8, 10, 9 (**A**); *n* = 10, 12, 7, 6 (**B**); *n* = 12, 12, 10, 10 (**C**); *n* = 11, 8, 6, 5 (**D**); *n* = 7, 9, 7, 9 (**E**); *n* = 11, 12, 7, 6 (**F**); *n* = 6 per group (**G**–**I**); and *n* = 8, 7, 7, 9 (**J**). All data are represented as mean ± SEM. *P* < 0.05 by 1-way ANOVA with Bonferroni’s corrections for multiple comparisons.

In sham-surgery rats, dapagliflozin treatment led to an approximately 100% increase in V_BDH_/V_CS_ and a 50%–80% reduction in both myocardial glucose uptake and V_PDH_/V_CS_ (Figure 5, A–F). Heart-failure rats did not demonstrate significant changes in baseline LV substrate utilization in the ischemic/infarction area, which is distal to the coronary artery ligation and includes border zones that have residual perfusion and metabolically active cardiomyocytes (51), as compared with sham-surgery rats (Figure 5, A–C). Heart-failure rats also had no change in substrate utilization in the nonischemic LV myocardium (Figure 5, D–F), which undergoes hemodynamic stress and remodeling, but is remote from the infarction. However, dapagliflozin treatment increased the relative rates of myocardial βOHB oxidation and decreased the relative rates of pyruvate oxidation in both regions in the heart-failure rats compared with vehicle control rats (Figure 5, B, C, E, and F).

Interestingly, the reduction in V_PDH_/V_CS_ was mirrored by a drop in glucose uptake in dapagliflozin-treated sham hearts, but not in the ischemic/infarct region of the heart-failure rats (Figure 5A). This suggests that there was an uncoupling of myocardial glucose oxidation and glucose uptake in the ischemic/infarct region, which likely reflected a shift to anaerobic glycolysis, in contrast to the nonischemic remote region where myocardial glucose uptake paralleled the reduction in V_PDH_/V_CS_ (Figure 5D). In dapagliflozin-treated compared with vehicle-treated heart-failure rats, the calculated rates of myocardial V_FA+AA_/V_CS_ were numerically higher in both the ischemic/infarct and nonischemic regions (Supplemental Figure 4, D and E), which paralleled their increase in plasma NEFA during SGLT2 inhibition. Together, these data indicate that dapagliflozin shifts the substrate preference for oxidative metabolism throughout the failing heart toward ketone oxidation and possibly free fatty acid and amino acid oxidation relative to pyruvate oxidation (Supplemental Figure 4, C and D).

### Acute dapagliflozin treatment does not alter cardiac gene expression in rats with heart failure.

We next measured myocardial mRNA and protein content of key mediators of cardiac metabolism. We analyzed tissue from the nonischemic LV myocardium remote from the infarct, so as to avoid the heterogeneity of the ischemic/infarct region. As shown in Supplemental Figure 4, E–G, acute dapagliflozin treatment did not significantly alter the mRNA content of important cardiac transporters and enzymes involved in glucose, fatty acid, or ketone oxidation. Interestingly, despite dapagliflozin-mediated reductions in myocardial glucose uptake and the relative rates of pyruvate oxidation, there were no significant differences in GLUT1 or GLUT4 protein expression (Figure 5G) or phosphorylated PDH (pPDH), although pPDH^S232^/PDH tended to increase (Figure 5H). Acute dapagliflozin treatment also did not significantly alter BDH1 or SCOT protein expression (Figure 5I), suggesting that dapagliflozin-mediated myocardial βOHB oxidation may be driven primarily by circulating βOHB levels, consistent with our previous observations in normal chow-fed rats. Similarly, the relative rates of βOHB oxidation positively correlated with circulating βOHB concentrations in both sham-surgery and heart-failure rats (Figure 5J), further highlighting that substrate availability is an important determinant of βOHB metabolism in heart failure.

### Acute dapagliflozin treatment does not significantly alter cardiac function or efficiency.

Recent evidence suggests that acute ketone supplementation can improve cardiac function and efficiency in the failing heart (22). To determine whether acute dapagliflozin-mediated increases in circulating plasma ketones and myocardial V_BDH_/V_CS_ impact heart function or cardiac efficiency in rats with heart failure, we performed echocardiography and metabolic analyses in a separate cohort of sham-surgery and heart-failure rats before and after acute dapagliflozin treatment. Two weeks after induction of MI or sham surgery, rats underwent baseline echocardiography. Heart-failure rats demonstrated significantly reduced LV ejection fraction, fractional shortening, and global longitudinal strain, while LV volumes also increased significantly, compared with sham-surgery controls (Supplemental Figure 5, A–G). Other parameters of systolic function (cardiac output and stroke volume) and diastolic function (E/e′) were also reduced in heart-failure rats compared with sham-surgery controls (Supplemental Figure 5, H–K).

**Figure 6:**
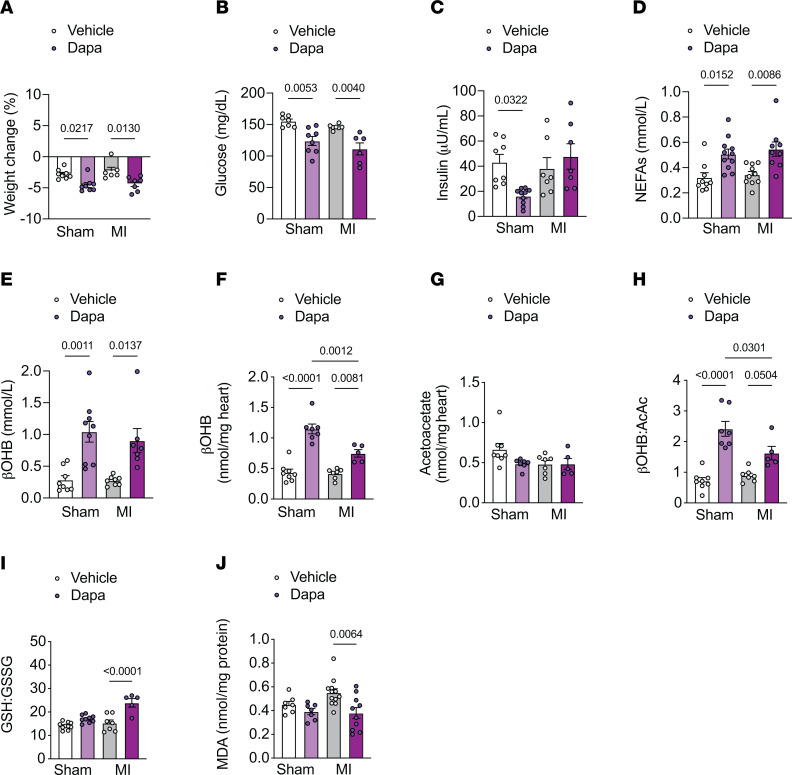
Acute dapagliflozin treatment increases plasma βOHB and improves mitochondrial redox in rats 2 weeks after MI. (**A**–**J**) Weight change (**A**), plasma glucose (**B**), plasma insulin (**C**), plasma NEFAs (**D**), plasma βOHB (**E**), myocardial βOHB (**F**), myocardial acetoacetate (**G**), myocardial βOHB:AcAc (**H**), myocardial GSH:GSSG (**I**) and myocardial malondialdehyde (MDA) (**J**) in chow-fed male rats 2 weeks after MI or sham surgery and treated with vehicle or 1.5 mg/kg body weight dapagliflozin (po) for 6 hours. In panels **A**–**J**, *n* = 8, 8, 6, 7 (**A**); *n* = 7, 8, 7, 6 (**B**); *n* = 8, 10, 7, 7 (**C**); *n* = 7, 9, 8, 7 (**D**); *n* = 6, 7, 6, 5 (**E**); *n* = 8, 7, 7, 5 (**F**–**H**); *n* = 10, 9, 7, 5 (**I**); and *n* = 7, 7, 12, 10 (**J**). All data are represented as mean ± SEM. *P* < 0.05 by 1-way ANOVA with Bonferroni’s corrections for multiple comparisons.

After confirming cardiac dysfunction and LV remodeling 2 weeks after coronary ligation, rats with heart failure were randomized to treatment groups. Rats were fasted the morning following the echocardiogram and treated with dapagliflozin or vehicle. A second echocardiogram was performed 6 hours after initiation of treatment. Body weight and fasting plasma glucose and insulin were lower in the dapagliflozin-treated groups compared with vehicle controls; dapagliflozin treatment also markedly increased fasting plasma NEFAs and circulating βOHB levels in sham-surgery and heart-failure rats, indicative of SGLT2 inhibition (Figure 6, A–E). Analysis of the echocardiographic data revealed no differences in the LV ejection fraction, global longitudinal strain (GLS), end-diastolic or end-systolic volume, stroke volume, or diastolic function (E/A, E/e′) with acute dapagliflozin treatment in either the heart-failure or sham-surgery control groups (Supplemental Figure 6, A–G). These results indicate that short-term dapagliflozin treatment does not acutely increase LV contractile function in rats with heart failure, despite the increase in plasma ketone levels and myocardial ketone oxidation. They also indicate that the metabolic changes that were observed with acute dapagliflozin treatment were not secondary to alterations in cardiac contractile function. Although the rats developed glucosuria during dapagliflozin treatment, there was not enough intravascular volume depletion to cause either a decrease in preload sufficient to measurably reduce the mitral E/e′ (an indicator of left atrial pressure) or the LV diastolic volume (Supplemental Figure 6, C and H).

### Acute dapagliflozin treatment alters mitochondrial redox and reduces markers of oxidative stress in heart-failure rats.

Myocardial ketone oxidation has previously been postulated to provide an additional source of energy to the failing heart and improve mitochondrial respiratory dynamics (15, 49). In order to test this hypothesis, we next assessed mitochondrial redox (βOHB:AcAc) and the content of short-chain CoA esters and adenine nucleotides in the noninfarcted myocardium in heart-failure rats acutely treated with dapagliflozin or vehicle for 6 hours. Similar to our chow-fed studies, acute dapagliflozin treatment served as a strong reductant of the mitochondrial nicotinamide adenine dinucleotide (NAD) system, as shown by the increased ratio of myocardial βOHB to acetoacetate in the failing LV myocardium (Figure 6, F–H). Intriguingly, the increase in LV mitochondrial reducing equivalents (βOHB:AcAc, a surrogate for NADH:NAD^+^) was independent of changes in myocardial acetyl-CoA and malonyl-CoA (Supplemental Figure 6, I and J), suggesting that it may be driven by increases in hepatic ketogenesis and circulating βOHB concentrations (47). An increase in cardiac mitochondrial reducing equivalents would be expected to (a) improve energy transfer and ATP production (15) and/or (b) contribute to the production of mitochondrial NAD(P)H, through nicotinamide nucleotide transhydrogenase, for the glutathione (GSH) redox cycle (52). Although acute dapagliflozin treatment did not alter cellular energy change (ATP:ADP, ATP:AMP) (Supplemental Figure 6, K and L), myocardial GSH: glutathione disulfide (GSSG) was increased in the LV myocardium of heart-failure rats with acute dapagliflozin treatment compared with vehicle-treated controls (Figure 6I). In addition, myocardial lipid peroxidation, a marker of oxidative stress, was reduced with acute dapagliflozin treatment in rats with heart failure (Figure 6J). Together, these data suggest that acute dapagliflozin-mediated increases in myocardial ketone oxidation and reducing equivalents are associated with reduced oxidative stress in rats with heart failure and are consistent with previous studies reporting the beneficial effects of SGLT2i to reduce oxidative stress (53–56).

### Chronic dapagliflozin treatment alters relative rates of myocardial substrate oxidation and mitochondrial redox and improves LV ejection fraction in heart-failure rats.

**Figure 7:**
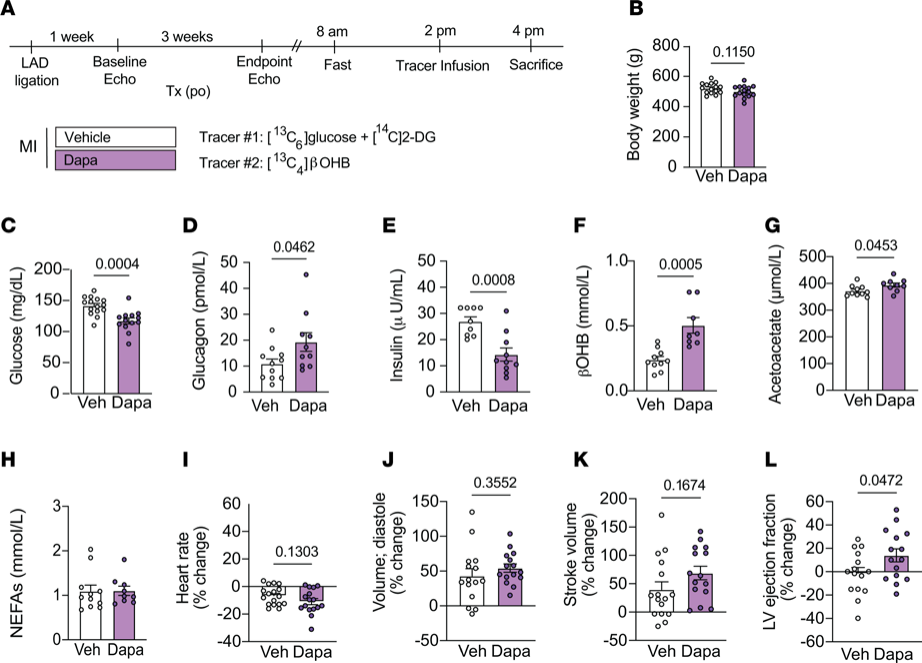
Chronic dapagliflozin treatment increases plasma β**OHB and improves LV ejection fraction in rats 4 weeks after MI.** (**A**) Outline of study design. Rats were given an intravenous bolus of ^14^C[2-DG] during the last 20 minutes of the infusion. (**B**–**L**) Body weight (**B**), plasma glucose (**C**), plasma glucagon (**D**), plasma insulin (**E**), plasma βOHB (**F**), plasma acetoacetate (**G**), plasma NEFAs (**H**), percentage change in heart rate (**I**), percentage change in diastolic volume (**J**), percentage change in stroke volume (**K**), and percentage change in LV ejection fraction (**L**) in chow-fed male rats 4 weeks after MI surgery and dapagliflozin (po) treatment (1.0 mg/kg body weight × 3 weeks). Panels **B**-**H**, were endpoint measures (4 weeks post-MI), while panels **I**-**L** represent percentage change from baseline (1 week post-MI compared with 4-weeks post-MI). In panels **B**–**L**, *n* = 15, 15 (**B** and **I**–**L**); *n* = 15, 13 (**C**); *n* = 11,10 (**D**); *n* = 9, 10 (**E)**; *n* = 10, 8 (**F**); *n* = 10, 9 (**G**); and *n* = 11, 9 (**H**). All data are represented as mean ± SEM. *P* < 0.05 by unpaired Student’s *t* test.

**Figure 8:**
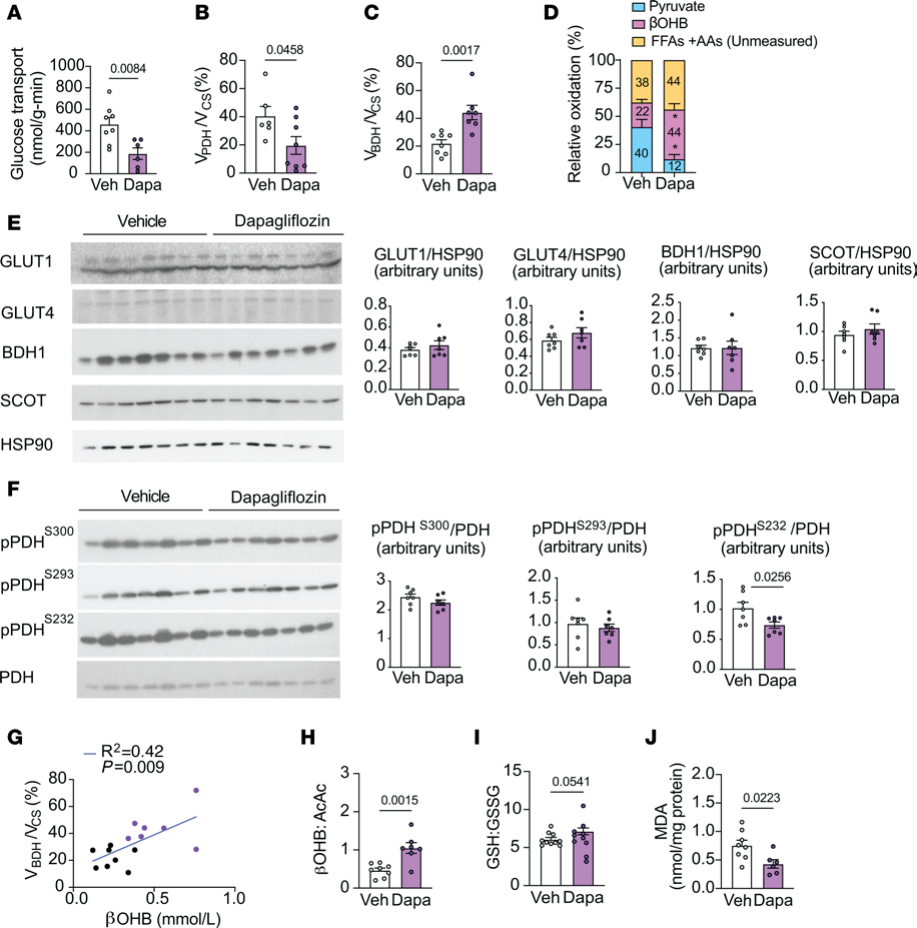
Chronic dapagliflozin treatment reduces and increases relative rates of glucose and βOHB oxidation, respectively, in the LV myocardium remote from the infarct area 4 weeks after MI. (**A**–**D**) Glucose uptake (**A**), relative rates of myocardial pyruvate oxidation (V_PDH_) to total mitochondrial oxidation (V_CS_) (**B**), relative rates of βOHB oxidation (V_BDH_) to total mitochondrial oxidation (V_CS_) (**C**), and relative rates of pyruvate, βOHB, and unmeasured free fatty acid and amino acid oxidation (**D**) in the nonischemic LV myocardium remote from the infarct area in chow-fed male rats 4 weeks after MI surgery and dapagliflozin (po) treatment (1.0 mg/kg body weight × 3 weeks). (**E**–**F**) Representative Western blot analysis of GLUT1, GLUT4, BDH1, and SCOT (**E**) and pPDH/PDH in the LV of MI rats treated as in **A**–**D**. *n* = 7 per group. HSP90 was used as a loading control. Quantification of blots shown in the right panels. (**G**) Correlation of plasma βOHB and V_BDH_/V_CS_ in rats treated as in **A**–**D**. (**H**–**J**) Myocardial βOHB:AcAc (**H**), myocardial GSH:GSSG (**I**), and myocardial MDA (**J**) in the LV of MI rats treated as in **A**–**D**. In panels **A**–**J**, *n* = 8, 6 (**A** and **J**); *n* = 6, 8 (**B**); *n* = 8, 7 (**C** and **G** and **H**); *n* = 6–8 per treatment group (**D**); *n* = 7, 7 (**E** and **F**); and *n* = 10, 10 (**I**). All data are represented as mean ± SEM. *P* < 0.05 by unpaired Student’s *t* test.

To determine whether the acute metabolic changes observed with SGLT2 inhibition were sustained and associated with enhanced cardiac function in the failing heart during more chronic dapagliflozin treatment, we next performed echocardiography and metabolic analyses in a separate cohort of heart-failure rats after 3 weeks of dapagliflozin treatment (~1 mg/kg body weight/day in drinking water) (Figures 7 and 8). One week after coronary artery ligation, male chow-fed rats underwent baseline echocardiography to confirm cardiac dysfunction and were randomized to treatment groups (Supplemental Table 2). A second echocardiogram was performed 3 weeks after dapagliflozin treatment, which was 1 day prior to metabolic assessment (Figure 7A). Notably, echocardiographic analyses revealed that chronic dapagliflozin treatment increased LV contractility, based on improvement in the LV ejection fraction, as compared with what occurred in vehicle-treated rats (Figure 7, I–L). There were no significant changes detected in LV volumes or heart rates (Figure 7, I–L).

We next assessed systemic and cardiac metabolic parameters. Body weight, fasting plasma glucose, plasma insulin, and the insulin/glucagon ratio were lower in chronic dapagliflozin-treated groups compared with vehicle controls; dapagliflozin treatment also increased plasma glucagon, whole-body βOHB turnover, whole-body glucose turnover, circulating βOHB, and circulating acetoacetate levels in heart- failure rats (Figure 7, B–G, and Supplemental Figure 7, A–C), indicative of SGLT2 inhibition. Interestingly, chronic dapagliflozin treatment did not further increase plasma NEFA concentrations (Figure 7H), which were already 2-fold higher than those observed in heart-failure rats 2 weeks after coronary artery ligation, possible due to higher catecholamine levels with long-term heart failure.

The myocardial metabolic analyses focused on the LV myocardium remote from the ischemic/infarct region, since at this later time point, the infarct zone expands and becomes progressively fibrotic, limiting its ability to provide insight into heart-failure metabolism. Dapagliflozin treatment led to an approximately 100% increase in relative rates of V_BDH_/V_CS_ and a 50% reduction in myocardial glucose uptake as well as the relative rate of V_PDH_/V_CS_ (Figure 8, A–C). The calculated myocardial V_FA+AA_/V_CS_ was numerically slightly higher in the dapagliflozin-treated compared with vehicle-treated heart-failure rats (Figure 8D). Consistent with our acute studies, dapagliflozin-mediated alterations in the relative rates of substrate utilization were not associated with appreciable differences in the protein expression of myocardial glucose transporters or proteins affecting βOHB oxidation (Figure 8E). While myocardial pPDH^S232^/PDH was decreased, no differences were detected in other inhibitory phosphorylation sites of PDH (Figure 8F). Relative rates of βOHB oxidation positively correlated with circulating βOHB concentrations in heart-failure rats (Figure 8G), suggesting that substrate availability remained an important determinant of βOHB metabolism in long-term heart failure.

Finally, we explored whether chronic dapagliflozin-mediated increases in myocardial ketone utilization might be associated with alterations in mitochondrial redox. As with acute inhibition, chronic SGLT2 inhibition increased plasma and myocardial mitochondrial redox (βOHB:AcAc) and myocardial GSH:GSSG and decreased myocardial lipid peroxidation (Figure 8, H–J, and Supplemental Figure 7, D–F). Taken together, these data indicate that chronic dapagliflozin treatment increases myocardial ketone oxidation and mitochondrial reducing equivalents, lowers oxidative stress, and improves LV ejection fraction in rats with heart failure.

## Discussion

The role of increased myocardial ketone oxidation in the cardioprotective effects of SGLT2 inhibition remains widely debated. Our study documents differential systemic and cardiac metabolic effects of dapagliflozin treatment versus exogenous ketone infusions in conscious animals, thus providing a fully integrated insight into the actions of dapagliflozin in vivo, which expands upon previously published reports (21, 23, 28, 33, 50, 53). We developed and utilized a stable isotope tracer method to directly assess the effects of acute and chronic SGLT2 inhibition on the relative rates of myocardial mitochondrial substrate utilization in a rat heart-failure model. We found that glucose and/or lactate-derived pyruvate was a major fuel source after a short fast and that ketone bodies contributed up to approximately 20% of total mitochondrial oxidation in the healthy rat myocardium. Acute dapagliflozin treatment increased hepatic ketogenesis and plasma βOHB availability and preferentially shifted mitochondrial myocardial metabolism toward ketone oxidation relative to pyruvate (glucose and lactate) oxidation in both the nonfailing and failing heart. Similar shifts in substrate utilization were sustained with more chronic dapagliflozin treatment in heart failure. Reductions in cardiac ketone utilization were observed when hepatic ketogenesis and plasma βOHB levels were reduced by altering the insulin-to-glucagon ratio by infusion of glucose in dapagliflozin-treated animals. Conversely, cardiac βOHB utilization was increased when animals were given an exogenous infusion of βOHB, further suggesting that cardiac ketone utilization is dependent on substrate availability, consistent with prior human studies (17, 57).

Unlike the well-defined substrate competition between fatty acids and glucose described by Randle (44), the effect of increased ketosis on cardiac fuel utilization is less clear (58). Prior studies have shown that myocardial ketone oxidation can supplement (24) or replace myocardial oxidation of fatty acids (59–61) or glucose (15, 62, 63). Here, we demonstrate that exogenous infusion of βOHB (plasma levels ~0.5–1 mM) increases relative cardiac βOHB utilization at the expense of fatty acid and amino acid oxidation in awake animals, while myocardial glucose uptake and the relative rates of pyruvate oxidation remain similar to those in saline-infused controls. Interestingly, the marked reduction in fatty acid oxidation occurred independently of changes in plasma NEFAs or cardiac malonyl-CoA content, the latter suggesting a CPT1-independent mechanism by which ketones inhibit fatty acid oxidation (59). The increase in myocardial acetyl-CoA content and mitochondrial redox during exogenous βOHB infusion suggests that ketones may inhibit fatty acid oxidation by altering the acetyl-CoA/free CoA ratio or NADH/NAD^+^ ratio (64).

In contrast, dapagliflozin treatment had very distinct systemic and cardiac metabolic effects compared with exogenous βOHB infusion. While both treatments increased the relative rates of myocardial ketone utilization, acute dapagliflozin had little effect on reducing fatty acid/amino acid oxidation and instead increased ketone oxidation at the expense of pyruvate oxidation. Differential actions of dapagliflozin and βOHB infusion on plasma hormones and NEFA levels were also observed, with dapagliflozin reducing plasma insulin and increasing plasma NEFA concentrations, in contrast to exogenous βOHB infusion. Together, these results highlight the importance of the extracardiac systemic effects of dapagliflozin on heart metabolism, beyond just elevating plasma ketone levels.

During the development of heart failure, myocardial fuel metabolism is reprogrammed, substantially impacting its progression (13, 65). Generally, it is believed that the failing heart is energetically compromised due to reductions in myocardial fatty acid (26) and glucose-derived pyruvate oxidation (25, 33, 66, 67), relying more on glycolysis (31, 68, 69). More recent in vivo and ex vivo studies in rodents and humans have shown that the failing myocardium has an increased capacity to utilize ketones (16–18, 24, 70), with variable effects on branched chain amino acid oxidation (21, 30, 71). Using a rodent model of heart failure induced by coronary artery ligation and MI (51), we evaluated cardiac substrate utilization 2 and 4 weeks after MI. Interestingly, rats with heart failure did not show significant changes in myocardial BDH1 expression or ketone utilization compared with sham-surgery rats. This lack of increased ketone utilization was likely attributed to unchanged hepatic ketogenesis and ketone availability in this model, consistent with prior studies showing similar ketone concentrations and cardiac ketone utilization in patients with heart failure (70, 72). Additionally, upregulation of ketone utilization may be related to the type of heart failure; for example, the model used in our study is less severe than another surgical heart- failure model that combines pressure overload and MI (73–75). Collectively, these data underscore the variability in circulating ketone availability, myocardial BDH1 protein expression, and myocardial ketone utilization that occur during heart failure (16–19, 21, 24, 28, 70, 76).

Myocardial glucose utilization and glycolysis have also been reported to be altered during the progression of heart failure (13). Specifically, myocardial glucose uptake and glycolysis are augmented in patients with heart failure with reduced ejection fraction (68, 77). Increases in myocardial glucose uptake have also been reported in experimental heart failure, such as canine models of cardiac pacing and Dahl salt-sensitive rats (78, 79). Although we did not assess glycolysis directly, our data using [^14^C]2-DG demonstrate that myocardial glucose uptake and phosphorylation as well as the relative rates of pyruvate oxidation are unaltered in both the ischemic/infarction area (which includes the border zone with residual metabolic activity; ref. 51) and the hemodynamically stressed nonischemic LV myocardium remote from the infarct. Since flow in the ischemic area is reduced in this model (51), the preserved overall myocardial glucose uptake in the ischemic area indicates an increase in fractional glucose extraction due to augmented glucose transport and glycolysis that occurs in ischemia (80).

Previous studies demonstrate that enhanced cardiac ketone delivery and utilization, achieved either through SGLT2 inhibition or exogenous ketone infusion, can improve cardiac function and efficiency in the failing heart (15, 20–22, 24, 81). Here, we observed that acute and chronic dapagliflozin treatments both markedly increased myocardial ketone oxidation, but only improved LV ejection fraction in heart-failure rats after chronic therapy. To our knowledge, acute SGLT2 inhibition has not been shown to have rapid effects on cardiac contractility or LV ejection fraction. In contrast, acute increases in LV ejection fraction have been reported in the literature during short term βOHB infusion in heart failure patients (22). However, these increases were minor at lower doses that achieved comparable plasma βOHB concentrations to those measured during dapagliflozin or ketone infusions in the current study. It is also noteworthy that acute ketone infusion had a significant and dose-dependent vasodilatory effect in heart failure patients, which contributed to their increased cardiac output (22). Although SGLT2i have less vasodilatory effects than high-dose ketone infusions, mild vasodilation might also contribute to an improvement in LVEF with chronic SGLT2i therapy in heart failure.

Aside from providing ancillary fuel for substrate metabolism, ketone bodies have additional cardioprotective effects, including reductions in oxidative stress (19, 82, 83). Our study indicates that dapagliflozin has chronic cardioprotective effects to reduce oxidative stress by increasing myocardial mitochondrial redox, presumably through cardiac ketone oxidation ensuing from increases in hepatic ketogenesis (48, 49). The NADH/NAD^+^ couple supports the divergent transfer of electrons from fuel substrates to both the electron transport chain (via complex I) for the generation of ATP and the antioxidant system via nicotinamide nucleotide transhydrogenase (NNT) (52). The observation that acute and chronic dapagliflozin treatment increased the GSH:GSSG ratio and lowered lipid peroxidation suggests that the excess NADH contributed to NADPH production for antioxidant defense (84). This antioxidant effect was evident in the absence of changes in cellular energy charge (ATP:ADP, ATP:AMP) or cardiac contractile function with acute SGTL2 inhibition in rats with heart failure. Indeed, a recent study demonstrated that βOHB supplementation led to a more reduced NAD(P)H/NAD^+^ redox state and augmented respiratory efficiency in isolated cardiac mitochondria from mice (15). These results are also consistent with previous work showing that supplementation of βOHB can improve cardiac respiratory efficiency and proton-driving force in healthy dogs and rats (48, 49, 85). Collectively, these findings suggest that over time the cardioprotective effects of dapagliflozin are due to a combination of reductions in oxidative stress and perhaps increased respiratory efficiency, both of which we propose are driven by increases in myocardial ketone oxidation and mitochondrial redox.

Our study demonstrates the differential effects of βOHB utilization versus SGLT2 inhibition on the relative rates of cardiac mitochondrial substrate utilization in conscious, unrestrained rodents. This approach avoided the confounding effects of anesthesia on the neurohumoral milieu and alterations in circulating hormones and metabolites that are key determinants of cardiac metabolism (86–88). However, there are several limitations worth discussing. First, our method relied on stable isotope tracing to track relative rates of pyruvate and βOHB oxidation and did not directly assess the absolute rates of oxidation. Thus, they indicate substrate preference for mitochondrial utilization, rather than quantitative rates of oxidative metabolism, and limited our ability to directly quantify the efficiency of ATP production and mitochondrial coupling, which are more readily assessed in working heart perfusions and isolated cardiac mitochondria (15, 24). Second, although we were able to estimate the relative rates of myocardial fatty acid oxidation by subtracting out pyruvate and βOHB oxidation rates, this calculation is not a direct measurement and slightly overestimates fatty acid utilization by the heart, since ketogenic amino acids also supply acetyl-CoA that enters the TCA cycle. Although ketogenic amino acids typically only contribute 1%–2% of cardiac energy production (13), recent studies have demonstrated that myocardial amino acid utilization is increased during heart failure (30). Thus, future studies using stable isotope tracer methodology targeted at amino acids are warranted to assess the effects of SGTL2 inhibition on cardiac ketogenic amino acid oxidation during heart failure. Third, the design of our study did not allow us to discern whether dapagliflozin-mediated reductions in the relative rates of pyruvate oxidation that we observed were due to decreases in glucose and/or lactate oxidation. However, cardiac glucose uptake tracked with relative rates of pyruvate oxidation in almost all of our infusion studies, suggesting that differences in pyruvate oxidation were determined at least in part by glucose-derived pyruvate. Finally, we assessed substrate oxidation in myocardial tissue, and while cardiomyocytes constitute the bulk of tissue mass, we cannot distinguish the contributions of different myocardial cell types to overall myocardial substrate oxidation. Intriguingly, it has recently been shown that both endothelial cells (89) and macrophages (90, 91) oxidize ketone bodies, suggesting that dapagliflozin’s metabolic and cardioprotective effects may also be determined in part by enhanced ketone oxidation in noncardiomyocyte cell types.

In conclusion, we utilized a [^13^C_4_]βOHB-labeling strategy to assess in vivo myocardial βOHB oxidation relative to total rates of mitochondrial oxidation in the intact rodent heart in conscious, unrestrained animals. Coupled with an infusion of [^13^C_6_]glucose, this strategy allowed us to rigorously assess the impact of acute SGLT2 inhibition on systemic metabolism as well as the relative rates of myocardial substrate utilization in healthy rats and rats with heart failure in a fully integrated neurohumoral milieu. Our results demonstrate that dapagliflozin treatment shifts myocardial substrate utilization toward ketone oxidation, at the expense of pyruvate rather than fatty acid oxidation. In marked contrast, ketone infusion reduced fatty acid oxidation, but had no effect on glucose uptake or pyruvate oxidation. Thus, the systemic effects of SGLT2i are pleiotropic and extend well beyond augmenting ketone availability. The fully integrated effects of dapagliflozin on circulating hormones and substrates are critical determinants of how SGLT2i regulate cardiac metabolism. This shift in substrate oxidation had acute and chronic cardioprotective metabolic effects to increase cardiac mitochondrial redox and reduce oxidative stress in rats with heart failure. However, recent studies have suggested that SGLT2i also have off-target effects. *Sglt2* knockout mice do not replicate the cardioprotective effects of SGLT2i, despite displaying similar metabolic profiles, and SGLT2i are protective against heart failure in *Sgtl2* knockout mice (92, 93). These latter findings highlight the need to better understand the degree to which shifts in cardiac metabolism versus off-target effects contribute to the beneficial action of SGLT2i treatment in human heart failure.

## Methods

Further information can be found in the Supplemental Methods (Supplemental Tables 3 and 4).

### Sex as a biological variable.

Our study exclusively examined male rats to minimize biological variability. It is unknown whether the findings are relevant for female rats.

### Statistics.

Animal sample size for each study was chosen to yield 90% power (at α = 0.05) to detect 20% differences in metabolic parameters, with an expected SEM of 15%. The number of animals used in each study is listed in the figure legends. Rodents were randomly allocated to experimental treatment groups based on LV ejection fraction for MI studies; weight matching was ensured before beginning experimental protocols. No outliers were excluded, and no experiments were terminated prematurely. In rare cases, animals were excluded due to technical issues (i.e., rats whose catheters did not remain patent after surgery, rats in the MI group who did not have a LV ejection fraction ≤ 45%, or rats who did not survive the LAD ligation surgery). Investigators were not blinded in all experiments due to practical reasons; however, ex vivo analyses of tissue samples were performed in a blinded fashion. Experimental values are represented as mean ± SEM. Data were analyzed using GraphPad Prism (GraphPad software, version 9); individual tests are described in the figure legends. *P* < 0.05 was considered statistically significant.

### Study approval.

All procedures were approved by the Institutional Animal Care and Use Committee of Yale School of Medicine and conducted in accordance with NIH guidelines.

### Data availability.

All data generated by the present study are included in this article and the Supplemental Data. Values for all data points in graphs are reported in the Supporting Data Values file.

## Author contributions

LG, LHY, and GIS conceived the project. LG, MK, GWC, LHY, and GIS developed methodology. LG, GWC, LHY, and GIS performed formal analysis. LG, YM, RCG, AN, JL, DZ, KDG, XH, JZ, NG, XL, TL, BTH, SH, XW, JS, SD, GMB, MK, and RJP performed experiments. LG, GWC, LHY, and GIS supervised the project. LHY and GIS provided resources. LG, LHY, and GIS acquired funding. LG created the figures and graphical abstract, which were edited by LHY and GIS. LG wrote the original draft of the manuscript. LG, LHY, and GIS reviewed and edited the manuscript with input from all authors.

## Supplementary Material

Supplemental data

Unedited blot and gel images

Supporting data values

## Version 1 12/16/2024

Electronic publication
