# Supplementary material for: SGLT2 inhibition alters substrate utilization and mitochondrial redox in healthy and failing rat hearts
Source: J Clin Invest. 2024 Dec 16;134(24):e176708. doi: 10.1172/JCI176708 (PMC11645152; doi:10.1172/JCI176708)
Supplement: Supplemental data [file jci-134-176708-s019.pdf]

SUPPLEMENTAL DATA

SGLT2 Inhibition Alters Substrate Utilization and Mitochondrial Redox in Healthy and Failing Rat Hearts

Leigh Goedeke[1,2,3,*], Yina Ma[4], Rafael Gaspar[1], Ali Nasiri[1], Jieun Lee[1], Dongyan Zhang[1], Katrine Galsgaard[1,5], Xiaoyue Hu[1], Jiasheng Zhang[1], Nicole Guerrera[1], Xiruo Li[1,6], Traci LaMoia[1,6], Brandon Hubbard[1,6], Sofie Haedersdal[1,7], Xiaohong Wu[1], John Stack[1], Sylvie Dufour[1], Gina Butrico[1], Mario Kahn[1], Rachel J. Perry[1,6], Gary W. Cline[1], Lawrence H. Young[4,6,*], and Gerald I. Shulman[1,6,*,#]

[1]Department of Internal Medicine (Endocrinology), Yale School of Medicine, New Haven CT, USA
[2]Department of Medicine (Cardiology) and The Cardiovascular Research Institute, Icahn School of Medicine at Mount Sinai, New York NY USA
[3]Department of Medicine (Endocrinology) and The Diabetes, Obesity and Metabolism Institute[4], Icahn School of Medicine at Mount Sinai, New York NY USA
[4]Department of Internal Medicine (Cardiovascular Medicine) and The Yale Cardiovascular Research Center, Yale School of Medicine, New Haven CT, USA
[5]Department of Biomedical Sciences, Faculty of Health and Medical Sciences, University of Copenhagen, Copenhagen, Denmark
[6]Department of Cellular & Molecular Physiology, Yale School of Medicine, New Haven CT, USA
[7]Department of Clinical Research, Copenhagen University Hospital, Steno Diabetes Center Copenhagen, Herlev, Denmark

[*]Correspondence: gerald.shulman@yale.edu, lawrence.young@yale.edu, leigh.goedeke@mssm.edu

[#]Lead contact: gerald.shulman@yale.edu

<u>This PDF file contains:</u>

Supplemental Methods
Supplemental References
Supplemental Tables 1–4
Supplemental Figures 1–7

Supplemental Methods

Animal studies. Seven-week old male Sprague Dawley (SD) rats were purchased from Charles River Laboratories (#001). Male SD rats were kept under constant temperature and humidity in a 12 hour controlled light/dark cycle (0700-1900 hours). All rats were allowed to acclimate to the Yale Animal Resource Center for one week prior to any experimental use. Rats were multiply housed prior to experimentation (2-3/standard cage) and singly housed after surgical procedures in individually vented cage racks with corncob bedding and shavings, gnawing blocks and Fat Rat Huts for enrichment. Chlorinated water was provided in an automatic watering system or water bottles. Rats were fed a standard chow diet (Harlan Teklad #2108S). One to four weeks prior to infusion studies, rats underwent surgery under isoflurane anesthesia to place polyethylene catheters (PE50/90 tubing, Instech Laboratories) in the carotid artery (infusion) and jugular vein (sampling). Carprofen analgesia was provided during the post-operative period and rats were allowed to recover for one week prior to performing experiments; only rats that recovered pre-surgery body weight were studied.

Heart failure was induced in male SD rats, utilizing a model of ischemic cardiomyopathy that develops after myocardial infarction caused by permanent ligation of the left anterior descending (LAD) coronary artery, as previously described (1). This model leads to substantial LV contractile dysfunction and progressive LV chamber enlargement in rats (1). Sham-treated rats underwent an identical surgical procedure as MI rats, but without coronary ligation (1). Echocardiography was performed under light isoflurane anesthesia one week or two weeks post-myocardial infarction to confirm baseline LV contractile dysfunction for chronic and acute dapagliflozin studies, respectively, as previously described (1). Rats were eligible for inclusion in the heart failure studies if baseline echocardiography confirmed an LV ejection fraction $\leq$45%. On the following day, qualifying rats with heart failure were randomized to treatment groups and underwent either 1) metabolic flux studies (stable isotope infusions after dapagliflozin or vehicle treatment; 2) acute cardiac function assessment (repeat echocardiography after dapagliflozin or vehicle treatment); or 3) chronic dapagliflozin treatment for three weeks followed by repeat echocardiography and metabolic flux studies. At the end of the studies, rats were sacrificed by intravenous pentobarbital (infusion studies) or isoflurane (basal studies) at 4 pm (6 hours following acute dapagliflozin treatment, 8 hours fasting). The heart LV myocardial tissue remote from the infarct (non-ischemic LV) and from the ischemic/infarct region of LV was immediately collected and freeze clamped with pre-cooled tongs in liquid N_2 and stored at –80°C until subsequent analyses. The ischemic/infarct region was excised from the LV anterior wall distal to the LAD suture occlusion extending to the apex. After in vivo coronary ligation, this region includes a central area of necrosis and the border zones, which have residual perfusion and residual cardiomyocytes that are still metabolically active (1).

For all acute SGLT2 inhibition studies, rats were fasted for 2 hours starting at 8 am and then treated with 1.5 mg/kg body weight dapagliflozin (p.o.) or vehicle (water) control (total volume: 3 ml/kg) at 10 am. Following treatment, drinking water was removed and rats were placed back in cages without food for an additional 4 hours, after which they were infused with $[^{13}C_6]$glucose or $[^{13}C_4]$D-βOHB for assessment of relative rates of myocardial substrate oxidation (see Metabolic flux studies) or subjected to echocardiography for basal cardiac function studies. In a certain set of experiments (“dapagliflozin + glucose”), dapagliflozin-treated rats were treated as above and simultaneously infused with glucose (variable infusion rate) to raise plasma glucose levels to that of vehicle controls. For βOHB infusion studies, rats were fasted as above and given an exogenous infusion of racemic D-L-βOHB (Sigma, 50 μmol/kg-min) for 2 hours. Control rats were infused with an equal amount of saline. Rats were sacrificed by intravenous pentobarbital (infusion studies) or isoflurane (basal studies) at 4 pm (6 hours following dapagliflozin treatment, 8 hours fasting). Tissue samples were immediately collected and freeze clamped with pre-cooled

tongs in liquid N_2 and stored at –80°C until subsequent analyses.

For all chronic SGLT2 inhibition studies, rats were randomized to treatment groups based on baseline echocardiography one week post-MI. Following randomization, rats were given drinking water containing 75 mg/L dapagliflozin (~1 mg/kg body weight based on water intake) for three weeks. Vehicle treated rats received drinking water with no dapagliflozin. One day prior to the metabolic infusions, rats underwent post-treatment echocardiography. The next morning (8 am) rats were placed in cages without food for 6 hours, after which they were infused with [$^{13}C_6$]glucose or [$^{13}C_4$]D-βOHB for assessment of relative rates of myocardial substrate oxidation (see Metabolic flux studies).

Echocardiography. Rats underwent anesthesia with (1-2%, v/v) isoflurane, and temperature was controlled and maintained with a heating pad. Echocardiography was performed as previously described (2) with a Visual-Sonics, Vevo 2100 instrument. 2D and M-mode images were obtained from parasternal short and long axis views. LV indexes of chamber diameter, area, injection fraction, fractional shortening, global longitudinal strain, stroke volume, and cardiac output were assessed.

Metabolic flux studies. Relative rates of cardiac-specific pyruvate oxidation (V_{PDH}) to total mitochondrial oxidation (V_{CS}) were measured after an 8 hour fast following a steady-state infusion with [$^{13}C_6$]glucose (1 mg/kg-min following a 5-min 3x prime) for 120 min. At the end of the infusion, whole blood was obtained from the venous catheter and immediately centrifuged and processed to plasma for the assessment of whole-body glucose turnover and plasma metabolites (see below). Rats were then sacrificed by an intravenous infusion of pentobarbital and tissues immediately (~15 seconds) excised and freeze-clamped in tongs pre-chilled in liquid N_2. Unless otherwise specified, frozen whole hearts were ground on dry ice and processed for analysis by LC-MS/MS and GC-MS to determine enrichments of [4,5-$^{13}C_2$]glutamate (m+2) and [$^{13}C_3$]alanine (m+3), respectively, for the calculation of myocardial V_{PDH}/V_{CS} flux as previously described (Eq. 1) (3-5):

$$(Eq. 1) \quad \frac{V_{PDH}}{V_{CS}} = \frac{[4,5-{}^{13}C_2]\text{glutamate}}{[{}^{13}C_3]\text{alanine}}$$

The ratio of [4,5-$^{13}C_2$]glutamate (m+2) and [$^{13}C_3$]alanine (m+3) reflects carbohydrate flux through V_{PDH} relative to V_{CS} (V_{PDH}/V_{CS}), such that a ratio of one indicates 100% pyruvate oxidation and any ratio less than one reflects dilution of the tracer from unlabeled acetyl-CoA (e.g. oxidation of fatty acids, ketones and/or ketogenic amino acids) (Supplemental Figure 1A). Although true V_{PDH}/V_{CS} flux requires measurement of [$^{13}C_2$]acetyl-CoA (m+2) and [$^{13}C_3$]pyruvate (m+3), isotopologues of acetyl-CoA and pyruvate in tissues from fasted animals undergo rapid degradation and are difficult to measure reliably due to differences in pool sizes (6, 7). We have previously shown that under conditions of steady-state, intracellular [$^{13}C_3$]pyruvate (m+3) equilibrates with [$^{13}C_3$]alanine (m+3) and intracellular [$^{13}C_2$]acetyl-CoA (m+2) equilibrates with [4,5-$^{13}C_2$]glutamate (m+2) in the muscles of fasted rodents (3, 5). A rigorous comparison study of the ^{13}C enrichments of these metabolites (performed in fasted rat hearts freeze-clamped *in situ* to minimize ischemic degradation) showed that these assumptions also hold true in heart muscle. As shown in Supplemental Figure 1, C and D, highly significant correlations were found between [$^{13}C_3$]alanine (m+3) and [$^{13}C_3$]pyruvate (m+3) (R^2=0.98, P = 0.0002) and [4,5-$^{13}C_2$]glutamate (m+2) and [$^{13}C_2$]acetyl-CoA (m+2) (R^2 = 0.89, P= 0.0042) over two different infusion rates, validating our equilibrium assumptions and indicating that [$^{13}C_3$]alanine and [4,5-$^{13}C_2$]glutamate enrichments could reliably be used to approximate V_{PDH}/V_{CS} in different excised heart regions

under fasting conditions. Myocardial alanine and pyruvate enrichments were measured by GC-MS (3, 8). Myocardial glutamate and acetyl-CoA enrichments were measured by LC-MS/MS (3).

Relative rates of heart βOHB oxidation (V_{BDH}) to total mitochondrial oxidation (V_{CS}) were measured in a separate cohort of rats after an 8 h fast following a steady-state infusion with [$^{13}C_4$]D-βOHB (0.1 mg/kg-min following a 5-min 3x prime) for 120 min. At the end of the infusion, whole blood was obtained from the venous catheter and immediately centrifuged and processed to plasma for the assessment of whole-body βOHB turnover and plasma metabolites. Rats were then sacrificed by an intravenous infusion of phenobarbital and tissues immediately excised and freeze-clamped in tongs pre-chilled in liquid N_2. Myocardial V_{BDH} /V_{CS} flux was measured using the following equation (Eq. 2):

$$(Eq. 2) \quad \frac{V_{BDH}}{V_{CS}} = \frac{[4,5-{}^{13}C_2]\text{glutamate}}{[{}^{13}C_4]\beta\text{OHB}}$$

The ratio of [4,5-^{13}C]glutamate (m+2) to that of [$^{13}C_4$]βOHB (m+4) reflects ketone flux of βOHB (V_{BDH}) relative to V_{CS} (V_{BDH} /V_{CS}), such that a ratio of one indicates 100% ketone oxidation and any ratio less than one reflects dilution of the tracer from unlabeled acetyl-CoA (e.g. oxidation of other substrates such as glucose, pyruvate, lactate, fatty acids and ketogenic amino acids) (Supplemental Figure 1H). Although true V_{BDH} /V_{CS} flux requires measurement of [$^{13}C_2$]acetyl-CoA (m+2), [4,5-$^{13}C_2$]glutamate (m+2) was used as a surrogate for [$^{13}C_2$]acetyl-CoA (m+2) due to the aforementioned difficulties in reliably measuring isotopologues of acetyl-CoA. As shown in Supplemental Figure 1K, a rigorous comparison of ^{13}C enrichments of these metabolites in *in situ* freeze-clamped hearts showed a significant correlation between [4,5-$^{13}C_2$]glutamate (m+2) and [$^{13}C_2$]acetyl-CoA (m+2) (R^2 = 0.85, P= 0.0241) over two different infusion rates, validating our equilibrium assumptions and indicating that [4,5-$^{13}C_2$]glutamate enrichments can reliably be used to approximate V_{BDH}/V_{CS} in the heart under fasting conditions. Myocardial βOHB enrichment was measured by GC-MS (9). Myocardial glutamate and acetyl-CoA enrichment was measured by LC-MS/MS (3).

The relative oxidation rate of fatty acids and ketogenic amino acids (V_{FFA+AA}) to total mitochondrial oxidation (V_{CS}) was indirectly assessed from relative rates of myocardial V_{BDH} /V_{CS} and V_{PDH} /V_{CS} obtained during the [$^{13}C_6$]glucose and [$^{13}C_4$]βOHB infusions as outlined in Eq. 3.

$$(Eq. 3) \quad \frac{V_{FFA+AA}}{V_{CS}} = 1 - \left(\frac{V_{BDH}}{V_{CS}} + \frac{V_{PDH}}{V_{CS}}\right)$$

Glucose uptake was measured in the heart, muscle and white adipose tissue of rats following a bolus injection of [^{14}C]2-deoxy-D-glucose during the last 20 minutes of the [$^{13}C_6$]glucose infusion. Briefly, freeze-clamped heart, muscle and white adipose tissue were processed to determine glucose uptake by comparing the plasma [^{14}C] specific activity decay curve to tissue [^{14}C] specific activity using a liquid scintillation counter (3).

Biochemical analyses. Blood samples were collected through venous catheters (infusion studies) or cardiac puncture (basal studies) after an 8 hour fast following treatment with dapagliflozin or vehicle control for 6 hours. Blood was immediately placed in heparinized-lithium tubes, separated by centrifugation, and stored at 4°C or –80°C for long-term storage. Plasma glucose concentrations were measured using a YSI Glucose Analyzer (Yellow Springs, OH), plasma insulin and glucagon by ELISA (Mercodia), and plasma βOHB by COBAS (Roche Diagnostics). Plasma NEFAs and acetoacetate were measured calorimetrically using kits from Wako Diagnostics and Biovision, respectively, according to the manufacturer's instructions.

Whole-body glucose and βOHB turnover was calculated in rats infused with $[^{13}C_6]$glucose or $[^{13}C_4]$D-βOHB for 2 hours. Turnover rates were determined using the following equation (Eq. 4), where APE denotes the atom percent enrichment determined by GC-MS as we have previously described (9).

$$(Eq. 4)\quad \text{Turnover} = \left(\frac{\text{Tracer APE}}{\text{Plasma APE}} - 1\right) \times \text{ Infusion rate}$$

βOHB clearance was calculated by dividing the measured βOHB turnover rate by plasma βOHB concentrations as previously described (9).

Urine samples were obtained directly from the bladder using a 20G needle immediately following sacrifice with intravenous pentobarbital. Urine βOHB and glucose were measured by COBAS (Roche Diagnostics) and a YSI Glucose Analyzer (Yellow Springs, OH), respectively.

Tissue Metabolite Measurements. Myocardial acetyl-CoA and malonyl-CoA concentrations were measured by LC-MS/MS as previously described (10). Myocardial βOHB and acetoacetate content were measured using kits from Sigma and Biovision, respectively, according to the manufacturers' instructions. Myocardial pyruvate and lactate concentrations were measured as previously described (11). Myocardial succinate, glutamate and malate concentrations were measured by LC-MS/MS using previously published methods (5). Myocardial ATP:AMP and ATP:ADP ratios were measured by LC-MS/MS as previously described (12).

Western Blot Analyses. Heart lysates were prepared in RIPA buffer with freshly added protease inhibitors (cOmplete MINI, Roche) and phosphatase inhibitors (PhosSTOP, Roche). After normalizing for equal protein concentrations by the BCA assay (Pierce), cell lysates were resuspended in Laemmli buffer sample buffer containing 4% 2-mercaptoethanol and separated on 4-8% Tris-Glycine Gels (Novex). Following a 1 hour semi-try transfer onto PVDF membranes, the membranes were blocked with 5% BSA (w/v) in TBST and probed with the indicated antibodies overnight at 4°C (Supplemental Table 3). After washing in TBST, membranes were incubated for 1 hour at RT with HRP-conjugated secondary antibodies (1:5,000) diluted in blocking buffer. Bands were visualized by enhanced chemiluminescence (Pierce) and densitometry analysis was carried out using ImageJ software (NIH).

RNA Isolation and qRT-PCR Analyses. Total rat tissue RNA was isolated using TRizol reagent (Invitrogen) and the RNEasy Mini Kit (Qiagen) according to the manufacturer's instructions. 1 μg of total RNA was reverse transcribed to cDNA using the QuantiTect Reverse Transcription Kit (Qiagen) and qPCR was performed in duplicate using iTaq SYBR Green (Bio-Rad) on an Applied Biosystems 7500 Fast qRT-PCR System. Relative gene expression was normalized to *Tbp*. See Supplemental Table 4 for primer sequences.

Oxidative Stress Measurements. Lipid peroxidation was determined in sham rats or rats with heart failure (two weeks post-MI or four weeks post-MI), following acute or chronic treatment with dapagliflozin or vehicle control using the TBARS Assay Kit (Cayman Chemical) as previously described (13). Myocardial glutathione redox state (GSH:GSSG) was determined by LC-MS/MS according to previously established methods (14).

Supplemental References

1. Pfau D, Thorn SL, Zhang J, Mikush N, Renaud JM, Klein R, et al. Angiotensin Receptor Neprilysin Inhibitor Attenuates Myocardial Remodeling and Improves Infarct Perfusion in Experimental Heart Failure. *Sci Rep.* 2019;9(1):5791.
2. Qi D, Atsina K, Qu L, Hu X, Wu X, Xu B, et al. The vestigial enzyme D-dopachrome tautomerase protects the heart against ischemic injury. *J Clin Invest.* 2014;124(8):3540-50.
3. Song JD, Alves TC, Befroy DE, Perry RJ, Mason GF, Zhang XM, et al. Dissociation of Muscle Insulin Resistance from Alterations in Mitochondrial Substrate Preference. *Cell Metab.* 2020;32(5):726-35 e5.
4. Wang Y, Nasiri AR, Damsky WE, Perry CJ, Zhang XM, Rabin-Court A, et al. Uncoupling Hepatic Oxidative Phosphorylation Reduces Tumor Growth in Two Murine Models of Colon Cancer. *Cell Rep.* 2018;24(1):47-55.
5. Perry RJ, Wang Y, Cline GW, Rabin-Court A, Song JD, Dufour S, et al. Leptin Mediates a Glucose-Fatty Acid Cycle to Maintain Glucose Homeostasis in Starvation. *Cell.* 2018;172(1-2):234-48 e17.
6. Alves TC, Befroy DE, Kibbey RG, Kahn M, Codella R, Carvalho RA, et al. Regulation of hepatic fat and glucose oxidation in rats with lipid-induced hepatic insulin resistance. *Hepatology.* 2011;53(4):1175-81.
7. Shulman GI, Rossetti L, Rothman DL, Blair JB, and Smith D. Quantitative analysis of glycogen repletion by nuclear magnetic resonance spectroscopy in the conscious rat. *J Clin Invest.* 1987;80(2):387-93.
8. LaMoia TE, Butrico GM, Kalpage HA, Goedeke L, Hubbard BT, Vatner DF, et al. Metformin, phenformin, and galegine inhibit complex IV activity and reduce glycerol-derived gluconeogenesis. *Proc Natl Acad Sci U S A.* 2022;119(10):e2122287119.
9. Perry RJ, Rabin-Court A, Song JD, Cardone RL, Wang Y, Kibbey RG, et al. Dehydration and insulinopenia are necessary and sufficient for euglycemic ketoacidosis in SGLT2 inhibitor-treated rats. *Nat Commun.* 2019;10(1):548.
10. Goedeke L, Bates J, Vatner DF, Perry RJ, Wang T, Ramirez R, et al. Acetyl-CoA Carboxylase Inhibition Reverses NAFLD and Hepatic Insulin Resistance but Promotes Hypertriglyceridemia in Rodents. *Hepatology.* 2018;68(6):2197-211.
11. LaMoia TE, and Shulman GI. Cellular and Molecular Mechanisms of Metformin Action. *Endocr Rev.* 2021;42(1):77-96.
12. Madiraju AK, Qiu Y, Perry RJ, Rahimi Y, Zhang XM, Zhang D, et al. Metformin inhibits gluconeogenesis via a redox-dependent mechanism in vivo. *Nat Med.* 2018;24(9):1384-94.
13. Goedeke L, Murt KN, Di Francesco A, Camporez JP, Nasiri AR, Wang Y, et al. Sex- and strain-specific effects of mitochondrial uncoupling on age-related metabolic diseases in high-fat diet-fed mice. *Aging Cell.* 2022;21(2):e13539.
14. Madiraju AK, Erion DM, Rahimi Y, Zhang XM, Braddock DT, Albright RA, et al. Metformin suppresses gluconeogenesis by inhibiting mitochondrial glycerophosphate dehydrogenase. *Nature.* 2014;510(7506):542-6.

Supplemental Table 1: Echocardiogram Parameters for Acute Study

Parameter[A]	Sham		MI	
	Vehicle	Dapagliflozin	Vehicle	Dapagliflozin
Heart rate (bpm)	339 ± 5	326 ± 7	324 ± 8	368 ± 31
Area; systole (mm^2)	40 ± 1	47 ± 4	73 ± 3*	77 ± 3*
Area; diastole (mm^2)	86 ± 2	83 ± 2	97 ± 3*	100 ± 4*
Volume; systole (μL)	134 ± 7	145 ± 8	372 ± 28*	394 ± 24*
Volume; diastole (μL)	505 ± 19	489 ± 17	632 ± 37*	661 ± 30*
Stroke Volume (μL)	371 ± 18	345 ± 60	265 ± 55*	267 ± 55*
Ejection Fraction (%)	73 ± 2	70 ± 1	42 ± 1*	41 ± 2*
Fractional Shortening (%)	42 ± 1	41 ± 1	17 ± 2*	16 ± 1*
Cardiac Output (mL/min)	122 ± 6	112 ± 5	84 ± 5*	89 ± 5*

[A]LV-trace parameters were measured two weeks post-MI one day prior to treatment with dapagliflozin (1.5 mg/kg body weight p.o.) or vehicle.

*P<0.05 compared to sham rats by one-way ANOVA with Bonferroni corrections for multiple comparisons. n = 15–17 per group. Data represent mean ± SEM.

Supplemental Table 2: Baseline Echocardiogram Parameters for Chronic Study

Parameter[A]	Vehicle	Dapagliflozin
**Heart rate (bpm)**	324 ± 7	320 ± 8
**Area; systole (mm^2)**	73 ± 3	73 ± 3
**Area; diastole (mm^2)**	97 ± 3	98 ± 4
**Volume; systole (μL)**	358 ± 26	360 ± 22
**Volume; diastole (μL)**	596 ± 32	596 ± 41
**Stroke Volume (μL)**	238 ± 17	236 ± 21
**Ejection Fraction (%)**	40 ± 2	39 ± 1
**Fractional Shortening (%)**	5 ± 1	8 ± 1
**Cardiac Output (mL/min)**	76 ± 5	69 ± 6

[A]LV-trace parameters were measured one week post-MI (baseline) prior to dapagliflozin (1 mg/kg body weight p.o.) or vehicle treatment for three weeks.

*P<0.05 compared to vehicle-treated rats by student's *t*-test. n = 15 per group.
Data represent mean ± SEM.

Supplemental Table 3: Antibody List

Antibody	Dilution	Source	Catalogue #
HSP90	1:1000	BD Biosciences	610419
BDH1	1:1000	Proteintech	15417-1-AP
SCOT	1:1000	Abcam	ab105320
PDHA1 (9H9AF51)	1:500	Abcam	ab110330
Phospho-PDH (Ser293)	1:500	Cell Signaling Technology	31866
Phospho-PDH (Ser232)	1:500	Millipore Sigma	AP1063
Phospho-PDH (Ser300)	1:500	Millipore Sigma	AP1064
GLUT1 (D3J3A)	1:500	Cell Signaling Technology	12939
GLUT4 (H-61)	1:500	Santa Cruz Biotechnology	sc-7938
GAPDH (14C10)	1:500	Cell Signaling Technology	2118
Anti-Rabbit IgG, HRP-linked	1:5000	Cell Signaling Technology	7074S
Anti-Mouse IgG, HRP-linked	1:5000	Cell Signaling Technology	7076S

Supplemental Table 4: Primer List

Gene	Forward	Reverse
Tbp	GGACTCCTGTCTCCCCTACC	CTCAGTGCAGAGGAGGGAAC
Sgtl2	CATTGTCTCAGGCTGGCACTGG	GGTGTTCATTGTGGCAGTGTCC
Sgtl1	ATGGACAGTAGCACCTTGAGCC	TAGCCCCAGAGAAGATGTCTGC
Glut4	CCGTGGCCTCCTATGAGATACT	AGGCACCCCGAAGATGAGT
Glut1	GCCTGAGACCAGTTGAAAGCAC	CTGCTTAGGTAAAGTTACAGGAG
Mpc1	AGATGAGTAAGCGGCCATCTGCCT	AGCCGAGAGTTGGTTTGGGGAT
Mpc2	TGCTGCCAAAGAAATTGAGGCCG	GCACAGTGGATTGAGCTGTGCTGA
Pdk4	GGATTACTGACCGCCTCTTTAGTT	GCATTCCGTGAATTGTCCATC
Mct1	GGCACCTCTTCTGGAATGCT	GCCCCTCAAACCCACACATA
Mct2	GGCCTTCGGTAGGATTAATAG	ATGCCTGATGATAACACGACT
Bdh1	TCCTGAGAAGGGAATGTGGG	AGTGAACTCCACCTCCCCAA
Scot1	CACCTTGCTACCCACTCCTG	CACAACCCGAAACCACCAAC
Acat1	AGCTCAAGACAGTGTTCCAGAA	GCTCCGTCGTTCAGTGTGC
Mcad	AGCCTTCACCGGATTCATCG	AGCCCCCATTGCAATCTTGA
Lcad	CCCTGGTTTCAGCCTCCATT	TCACTCCCAGACCTTTTGGC
Cpt1b	CATTTCCGGGACAAAGGCAAGT	CGTGGACTCGCTAGTACAGGAA
Fabp4	AGAACTCACTGGGACCTGGA	TCTCGGACAGCAATCAGCTC
Cd36	GCCTCCTTTCCACCTTTTGT	GATTCAAACACAGCATAGATGGAC

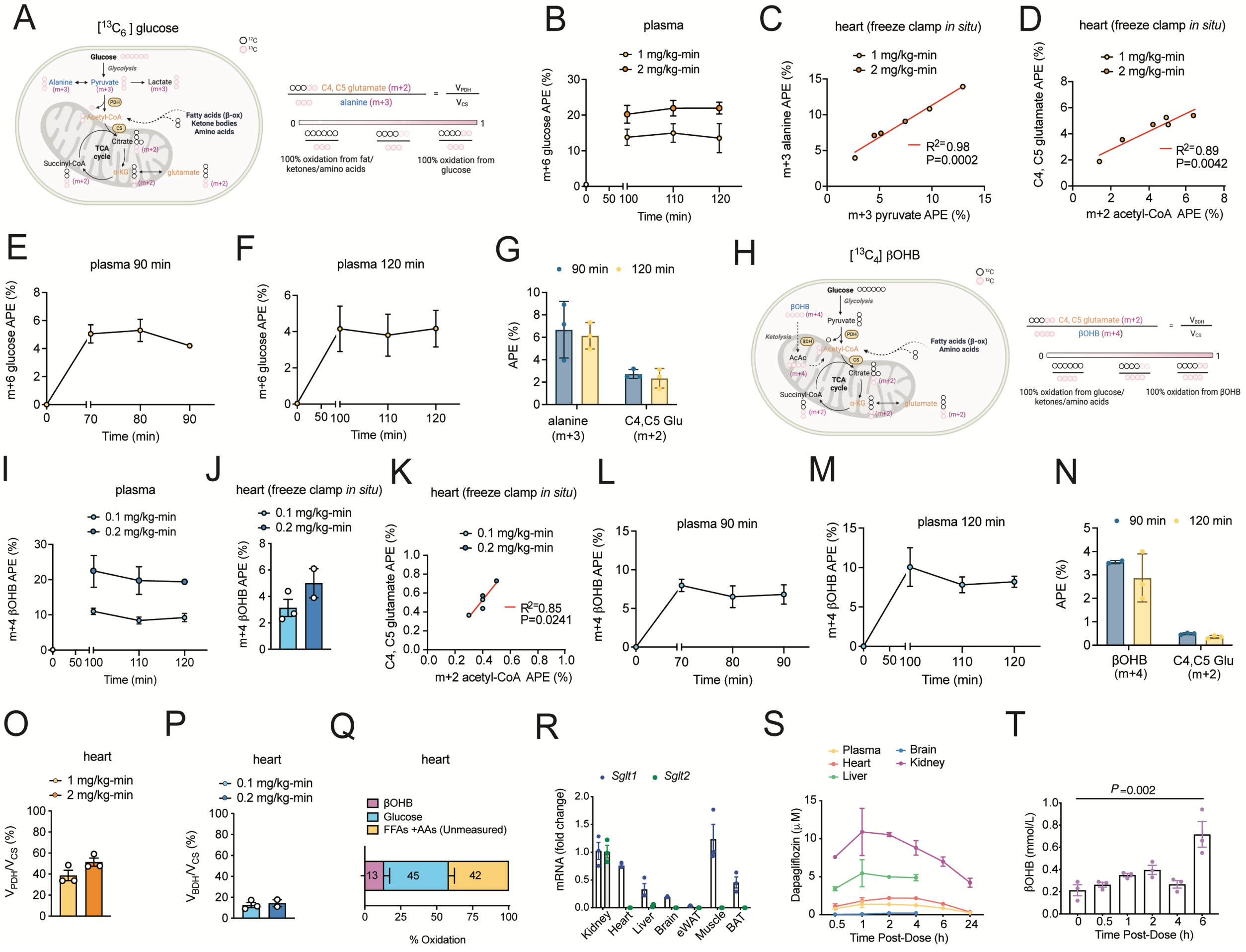

Supplemental Figure 1: A stable isotope approach to assess cardiac-specific relative rates of pyruvate and βOHB oxidation. (A) Relative rates of myocardial pyruvate oxidation were determined using GC-MS and LC-MS/MS methodology following a steady infusion of $[^{13}C_6]$glucose. Equations used to calculate V_{PDH}/V_{CS} are shown to the right. (**B**) Plasma $[^{13}C_6]$glucose (m+6) enrichments following a steady-state infusion of 1 or 2 mg/kg-min $[^{13}C_6]$glucose for 120 min. (**C**) Correlation of myocardial $[^{13}C_3]$alanine (m+3) and $[^{13}C_3]$pyruvate (m+3) enrichments. (**D**) Correlation of myocardial $[^{13}C_2]$acetyl-CoA (m+2) and $[4,5\text{-}^{13}C_2]$glutamate (m+2) enrichments. **(E–G**) Plasma $[^{13}C_6]$glucose (m+6) (**E–F**) and myocardial $[^{13}C_3]$alanine (m+3) and $[4,5\text{-}^{13}C_2]$glutamate (m+2) enrichments (**G**) in rats infused with $[^{13}C_6]$glucose for 90 or 120 min. (**H**) Relative rates of myocardial βOHB oxidation were determined using GC-MS and LC-MS/MS methodology following a steady infusion of $[^{13}C_4]$βOHB in chow-fed male rats. Equations used to calculate relative rates of V_{BDH}/V_{CS} are shown to the right. (**I**) Plasma $[^{13}C_4]$βOHB (m+4) enrichments following a steady-state infusion of 0.1 or 0.2 mg/kg-min $[^{13}C_4]$βOHB for 120 min. (**J**) Myocardial $[^{13}C_4]$βOHB (m+4) enrichments. (**J**) Correlation of myocardial $[^{13}C_2]$acetyl-CoA (m+2) and $[4,5\text{-}^{13}C_2]$glutamate (m+2) enrichments. (**L–N**) Plasma $[^{13}C_4]$βOHB (m+4) (**L–M**) and myocardial $[^{13}C_4]$βOHB (m+4) and $[4,5\text{-}^{13}C_2]$glutamate (m+2) enrichments (**N**) in rats infused with $[^{13}C_4]$βOHB for 90 or 120 min. (**O–P**) Relative rates of myocardial pyruvate (V_{PDH}) (**O**) and βOHB (V_{BDH}) (**P**) oxidation relative to total mitochondrial oxidation (V_{CS}). (**Q**) Relative rates of myocardial glucose, βOHB and fatty acid +ketogenic amino acid oxidation. Relative rates of fatty acid and

ketogenic amino acid oxidation were estimated using the following equation: $[FFA + AA_{OX}] = 1 - [V_{BDH}/V_{CS} + V_{PDH}/V_{CS}]$. (**R**) Tissue *Sglt1* and *Sglt2* mRNA expression. (**S**) Dapagliflozin concentrations following an oral dose of dapagliflozin (1.5 mg/kg body weight). (**T**) Plasma βOHB concentrations following oral dapagliflozin treatment. All data are mean ± SEM. *n* = 2–3 rats per group or timepoint. In panel (**T**), $*P<0.05$ by one-way Kruskal-Wallis test with Dunn's correction for multiple comparisons.

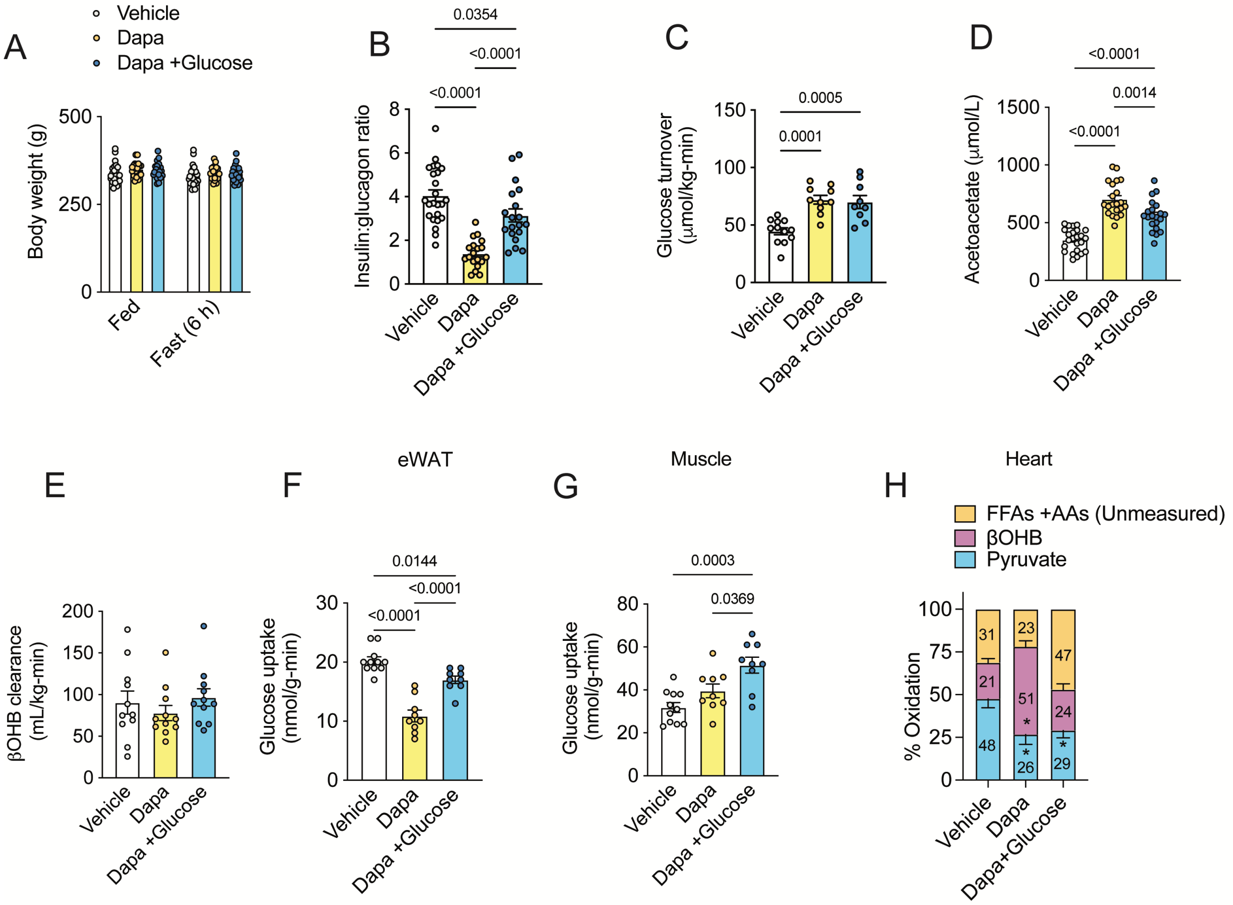

Supplemental Figure 2: Variable glucose infusion in acute dapagliflozin-treated rats increases glucose uptake in eWAT and muscle. (**A**–**H**) Pre-treatment body weight (**A**), insulin:glucagon ratio (**B**), whole-body glucose turnover (**C**), plasma acetoacetate (**D**), βOHB clearance (**E**), eWAT glucose uptake (**F**) muscle glucose uptake (**G**) and relative rates of myocardial substrate oxidation (**H**) in chow-fed male Sprague Dawley rats treated as in **Figure 1A.** n = 9–25 per group. All data are mean ± SEM. *P<0.05 by one-way ANOVA with Bonferroni corrections for multiple comparisons. eWAT: epidydimal white adipose tissue. Dapa, dapagliflozin.

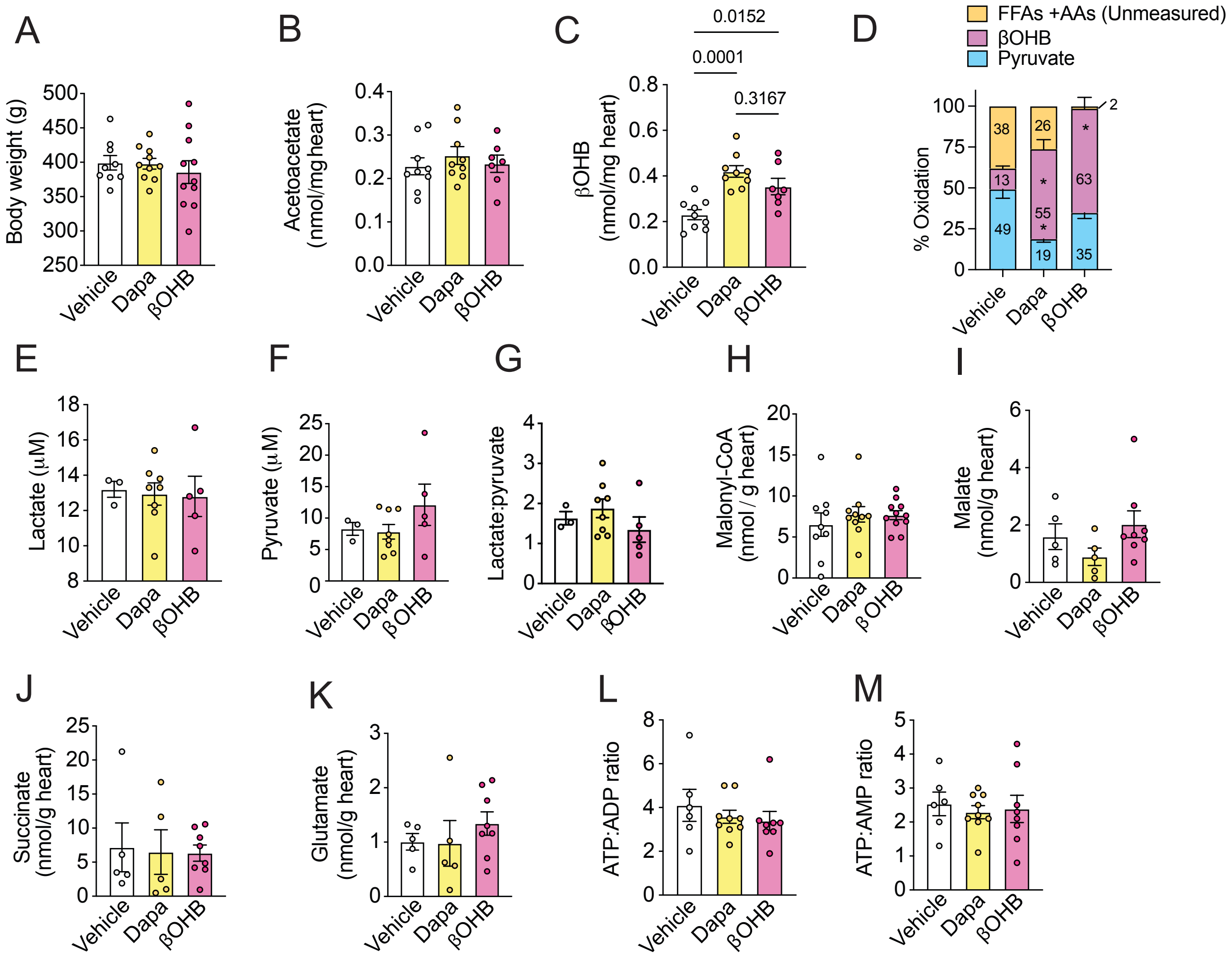

Supplemental Figure 3: Acute βOHB infusion increases myocardial βOHB oxidation independently of changes in cytosolic redox and anaplerosis. (**A**–**D**) Pre-treatment body weight (**A**), myocardial acetoacetate content (**B**), myocardial βOHB content (**C**) and relative rates of myocardial substrate oxidation (**D**) in the hearts of chow-fed male Sprague Dawley rats treated with vehicle, 1.5 mg/kg body weight dapagliflozin (p.o.) or infused with 50 µmol/(kg-min) βOHB. (**E**–**M**) Myocardial lactate (**E**), pyruvate (**F**), cytosolic redox (lactate:pyruvate) (**G**), malonyl-CoA content (**H**), malate content (**I**), succinate content (**J**), glutamate content (**K**), ATP:ADP ratio (**L**) and ATP:AMP ratio (**M**) in the hearts of rats treated as in (**A**). All data are mean ± SEM. *n* = 3–8 per treatment group. *P*<0.05 by one-way ANOVA with Bonferroni corrections for multiple comparisons. Dapa, dapagliflozin.

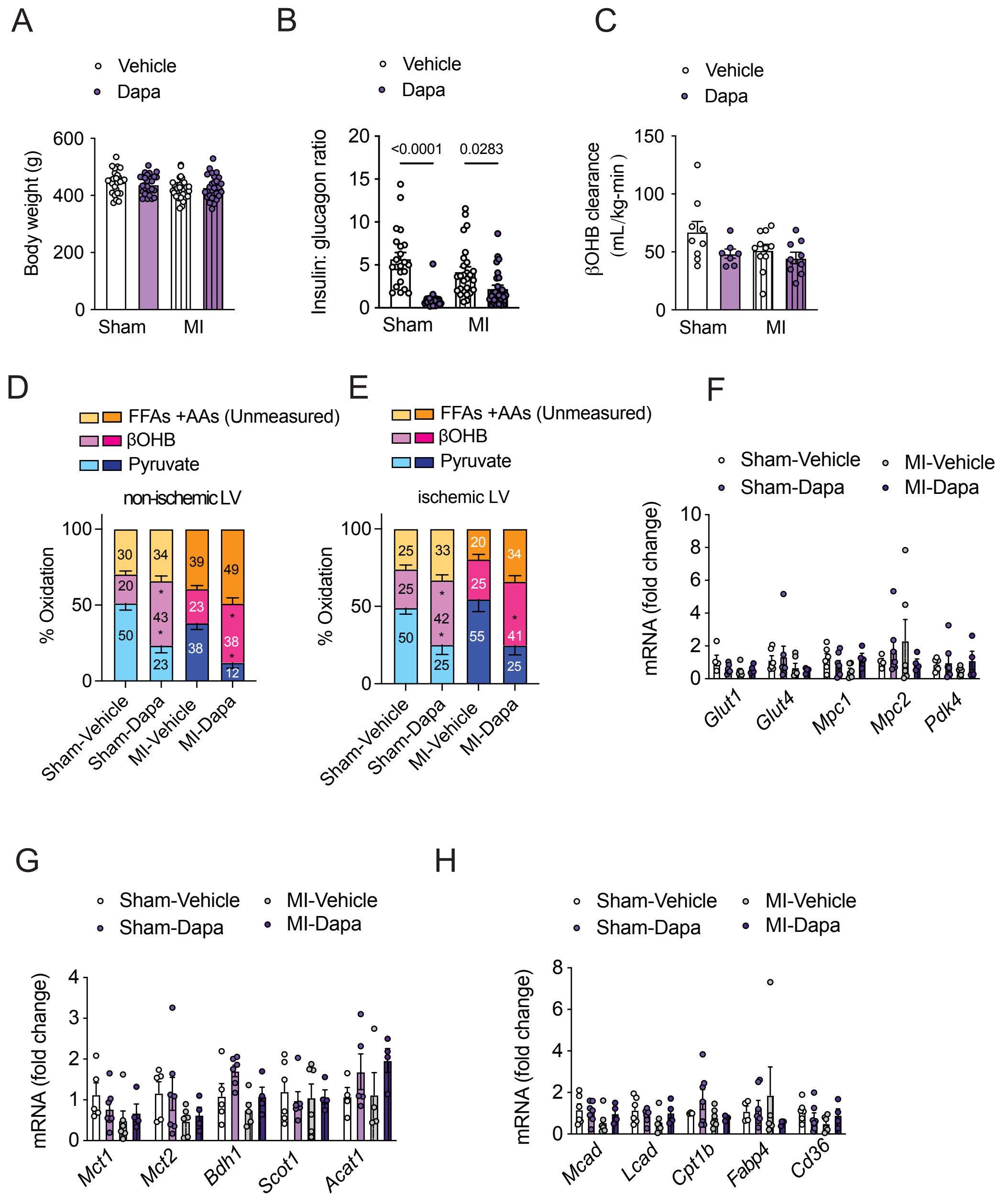

Supplemental Figure 4: Acute dapagliflozin treatment alters myocardial substrate oxidation in sham and heart failure rats. (**A**–**C**) Pre-treatment body weight (**A**), insulin:glucagon ratio (**B**) and βOHB clearance (**C**) in chow-fed male Sprague Dawley rats two-weeks post MI and sham surgery and acutely treated with dapagliflozin (1.5 mg/kg body weight) or vehicle control. (**D**–**E**) Relative rates of myocardial substrate oxidation in the non-ischemic left ventricular (LV) myocardium (**D**) and ischemic LV myocardium (**E**) in rats treated as in (**A**). (**F**–**H**) Myocardial mRNA expression of glucose uptake/oxidation genes (**F**), βOHB uptake/oxidation genes (**G**) and fatty acid uptake/oxidation genes (**H**) in the LV remote from the myocardium of chow-fed male Sprague Dawley rats two-weeks post-MI or sham surgery. *n* = 6–33 per group. All data are mean ± SEM. **P*<0.05 by one-way ANOVA with Bonferroni corrections for multiple comparisons. Dapa, dapagliflozin.

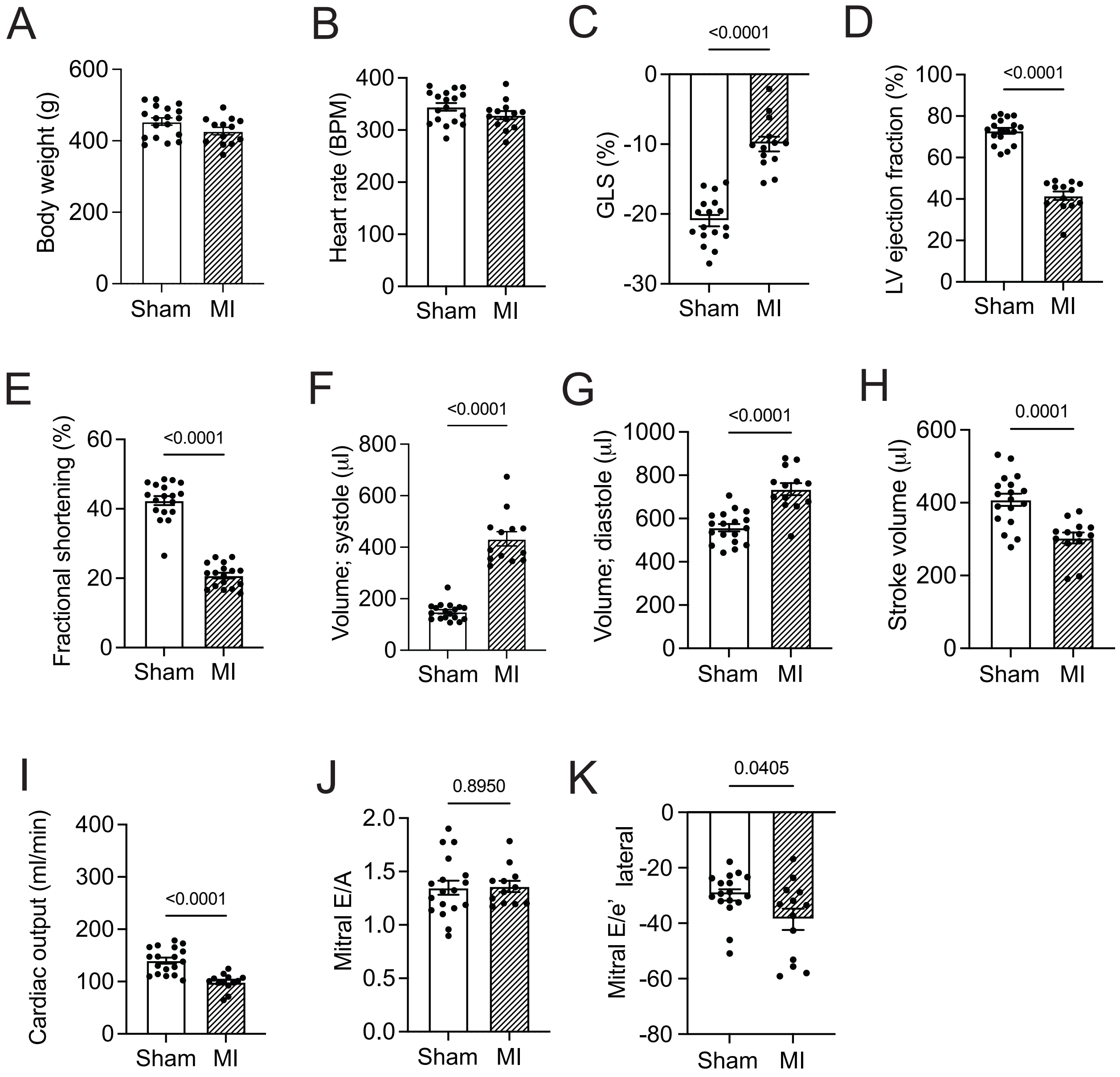

Supplemental Figure 5: LAD ligation induces LV dysfunction two-weeks post MI. (**A**–**K**) Body weight (**A**), heart rate (**B**), global longitudinal strain, GLS (**C**), LV ejection fraction (**D**), fractional shortening (**E**), systolic volume (**F**), diastolic volume (**G**), stroke volume (**H**), cardiac output (**I**), mitral E/A (**J**) and mitral E/e' (**K**) in male Sprague Dawley rats two-weeks post MI or sham surgery. *n* = 13–18 per group. All data are mean ± SEM. **P*<0.05 by unpaired students *t*-test.

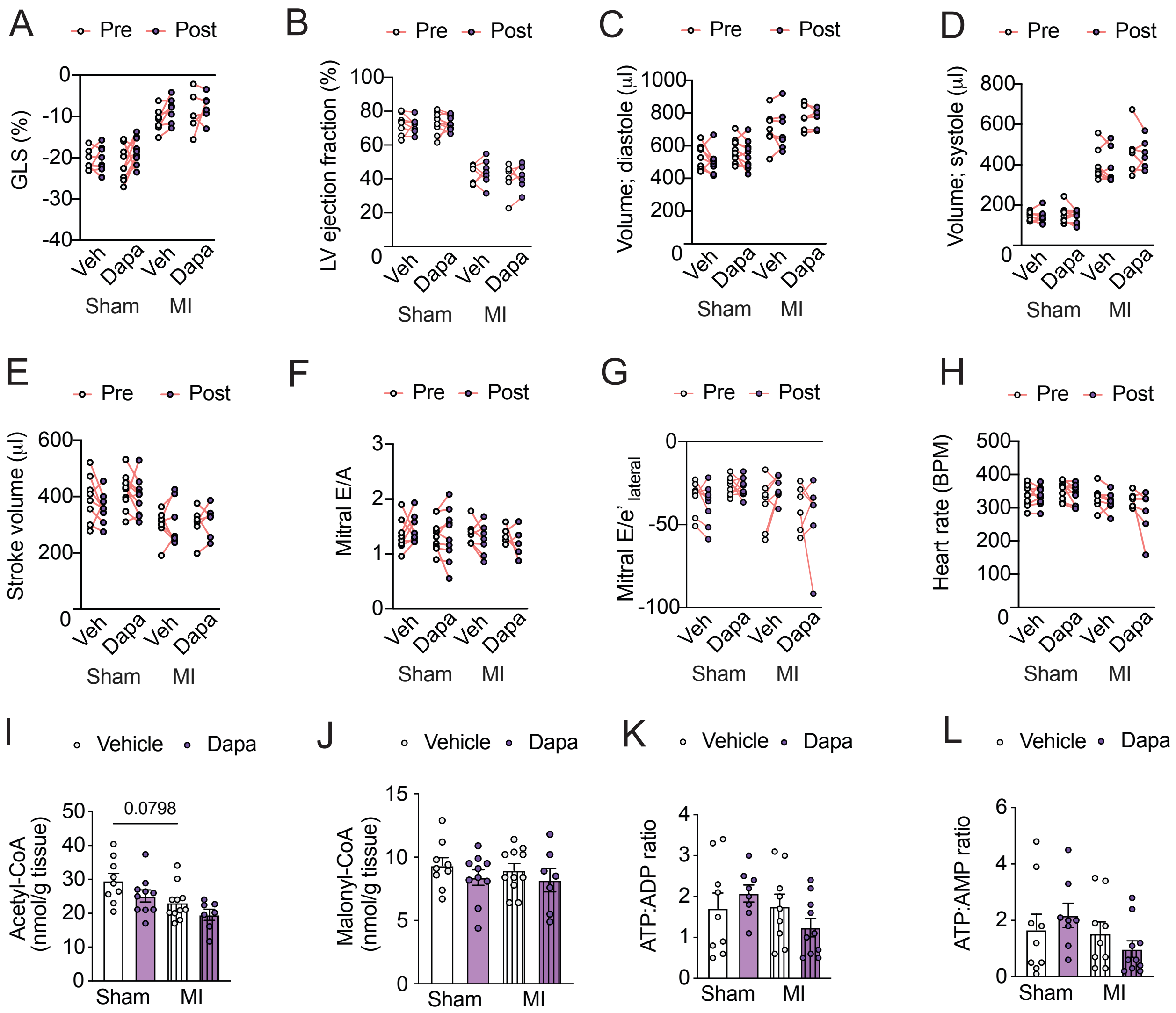

Supplemental Figure 6: Acute dapagliflozin treatment does not alter cardiac function in rats with heart failure. (A–L) Global longitudinal strain (GLS) (**A**), LV ejection fraction (**B**), diastolic volume (**C**), systolic volume (**D**), stroke volume (**E**), mitral E/A (**F**), mitral E/e' (**G**), heart rate (**H**), myocardial acetyl-CoA content (**I**), myocardial malonyl-CoA content (**J**), myocardial ATP:ADP ratio (**K**) and myocardial ATP:AMP ratio (**L**) in male Sprague Dawley rats two-weeks post MI or sham surgery acutely treated with dapagliflozin (1.5 mg/kg body weight, p.o.) or vehicle control for 6 hours. *n* = 6-10 per treatment group. All data are mean ± SEM. **P*<0.05 by paired student's *t*-test (**A–H**) or one-way ANOVA with Bonferroni corrections for multiple comparisons (**I–L**). Veh, vehicle; Dapa, dapagliflozin.

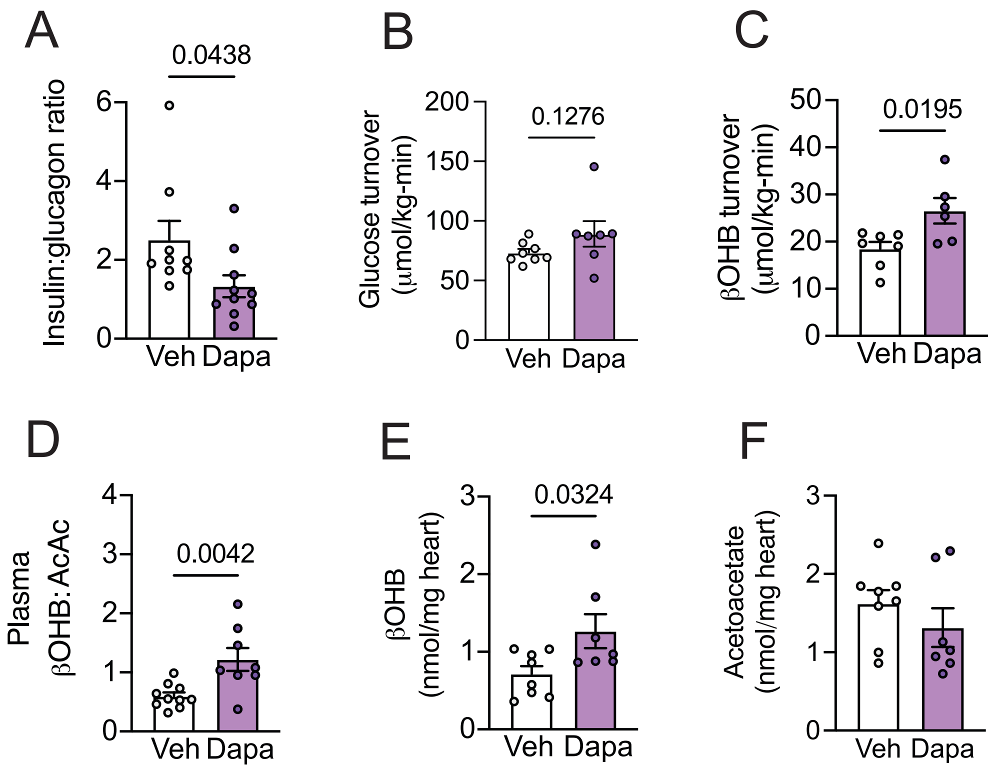

Supplemental Figure 7: Chronic dapagliflozin treatment reduces insulin:glucagon ratio and increases whole-body βOHB turnover in rats with heart failure. (A–F) Plasma insulin:glucagon ratio (**A**), whole-body glucose turnover (**B**), whole-body βOHB turnover (**C**), plasma mitochondrial redox (βOHB:AcAc) (**D**), myocardial βOHB content (**E**) and myocardial acetoacetate content (**F**) in male Sprague Dawley rats four-weeks post MI surgery and chronically treated with dapagliflozin (1 mg/kg body weight) or vehicle control for three weeks. n = 5-10 per treatment group. All data are mean ± SEM. *P<0.05 by unpaired student's t-test. Veh, vehicle; Dapa, dapagliflozin.
