# Supplementary material for: SGLT2 inhibition alters substrate utilization and mitochondrial redox in healthy and failing rat hearts
Source: J Clin Invest. 2024 Dec 16;134(24):e176708. doi: 10.1172/JCI176708 (PMC11645152; doi:10.1172/JCI176708)
Supplement: Unedited blot and gel images [file jci-134-176708-s020.pdf]

Uncropped Western Blots

176708-JCI-RG-RV-2 Goedeke *et al*

Figure 3F

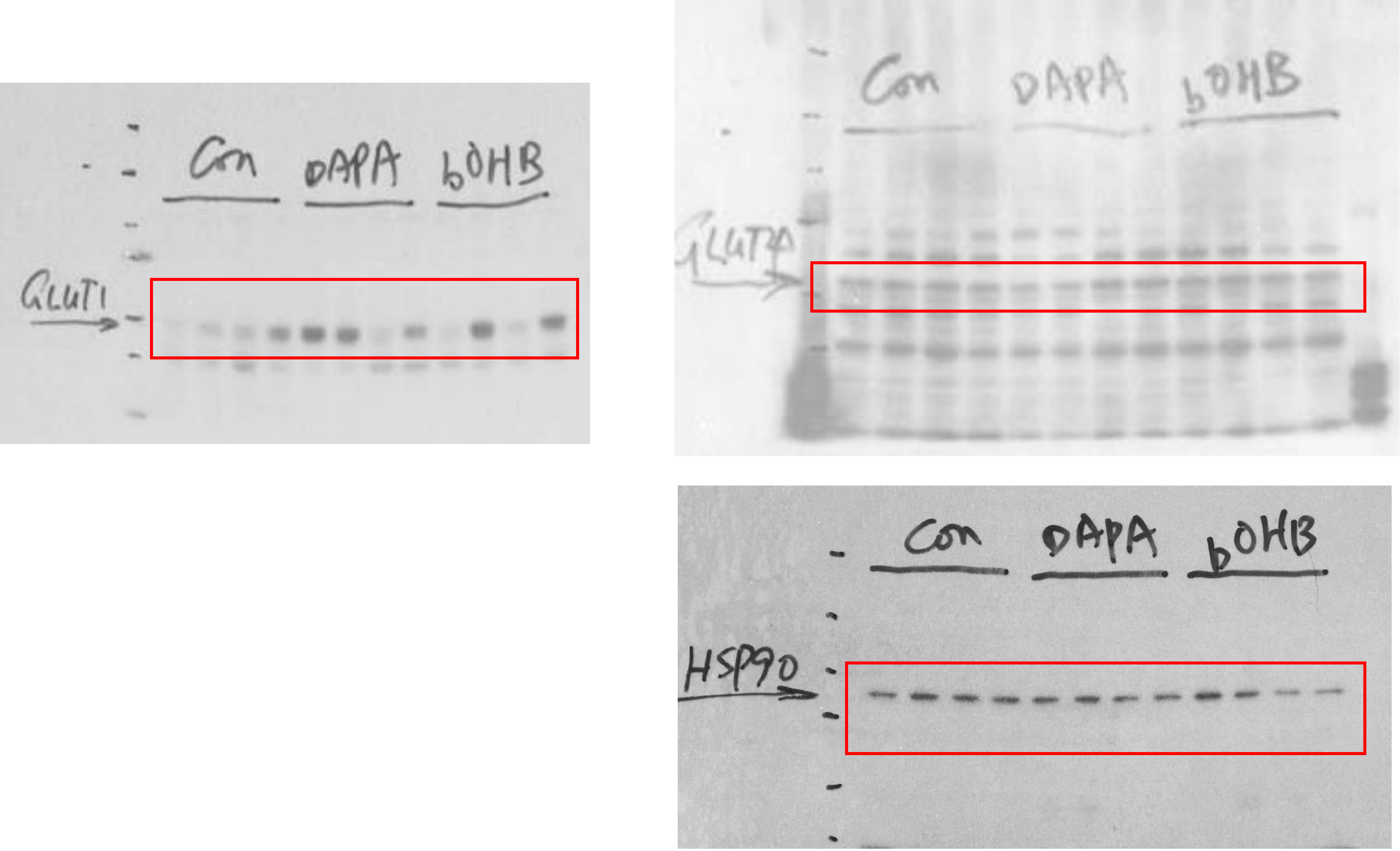

Figure 3G

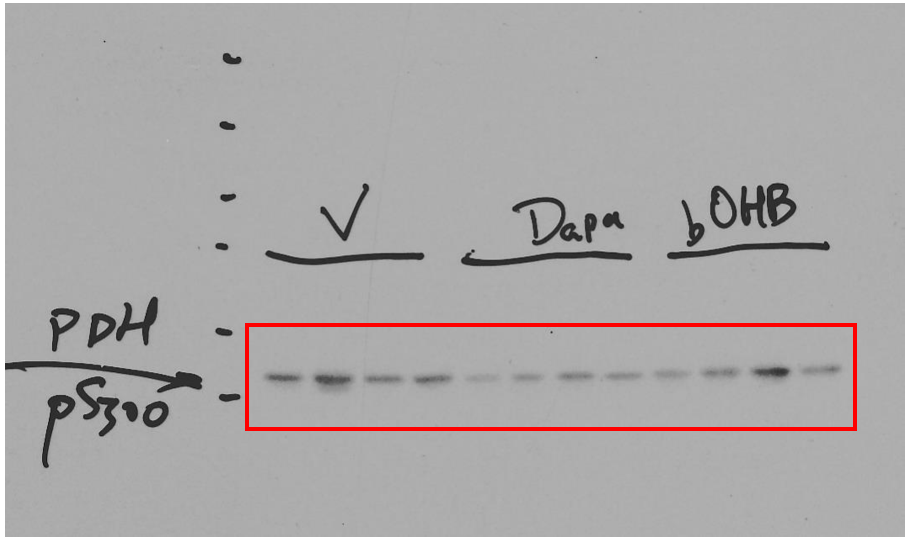
V
Dapa
bOHB
PDH
pS300

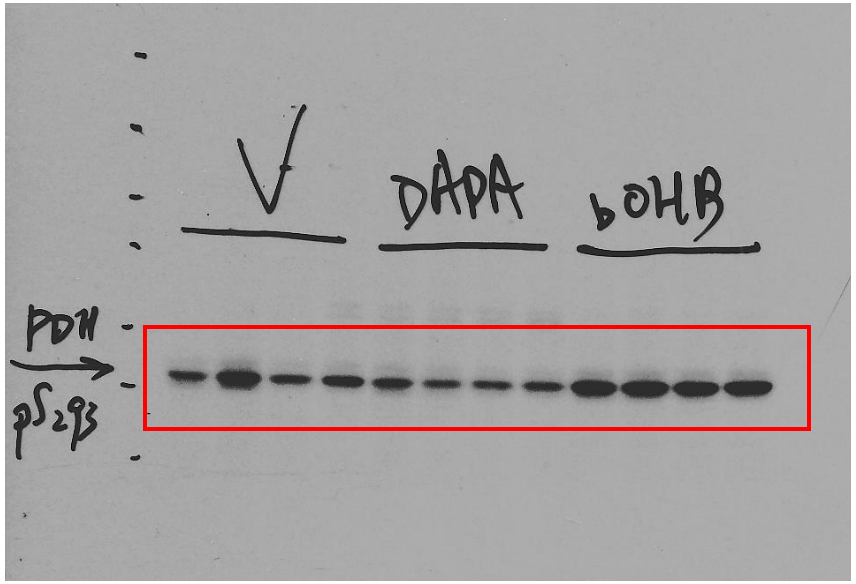
V
DAPA
bOHB
PDH
pS293

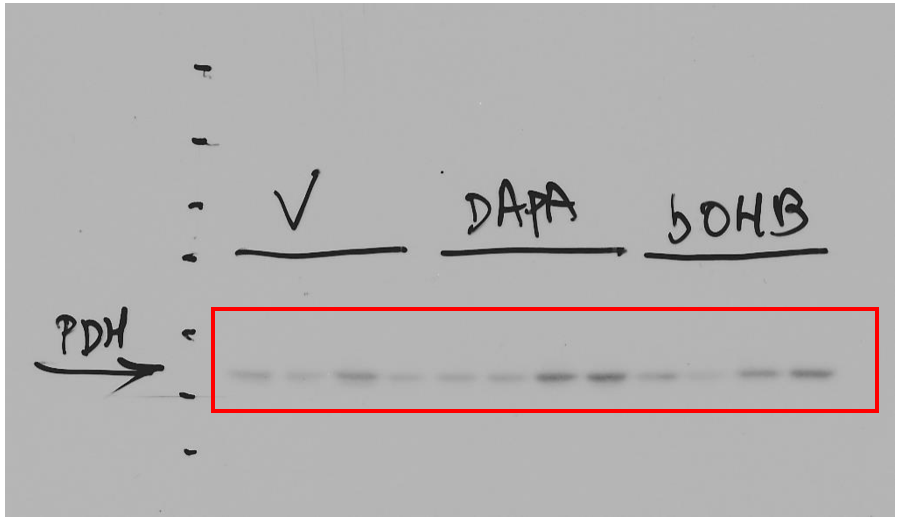
V
DAPA
bOHB
PDH

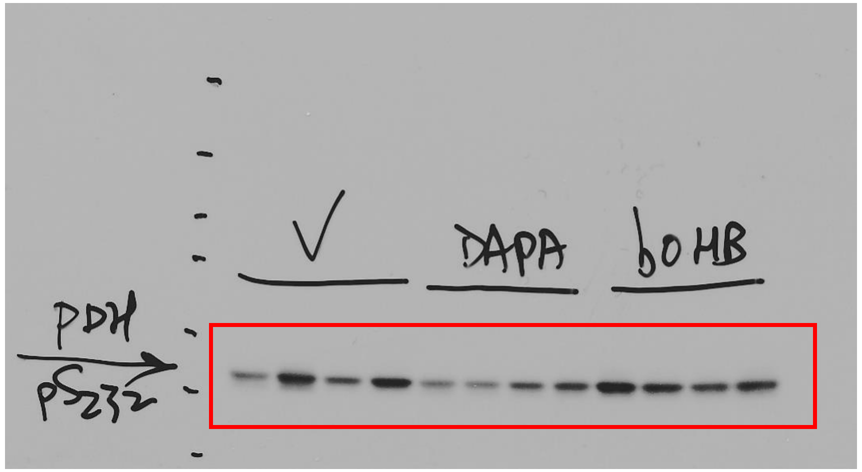
V
DAPA
bOHB
PDH
pS232

Figure 3H

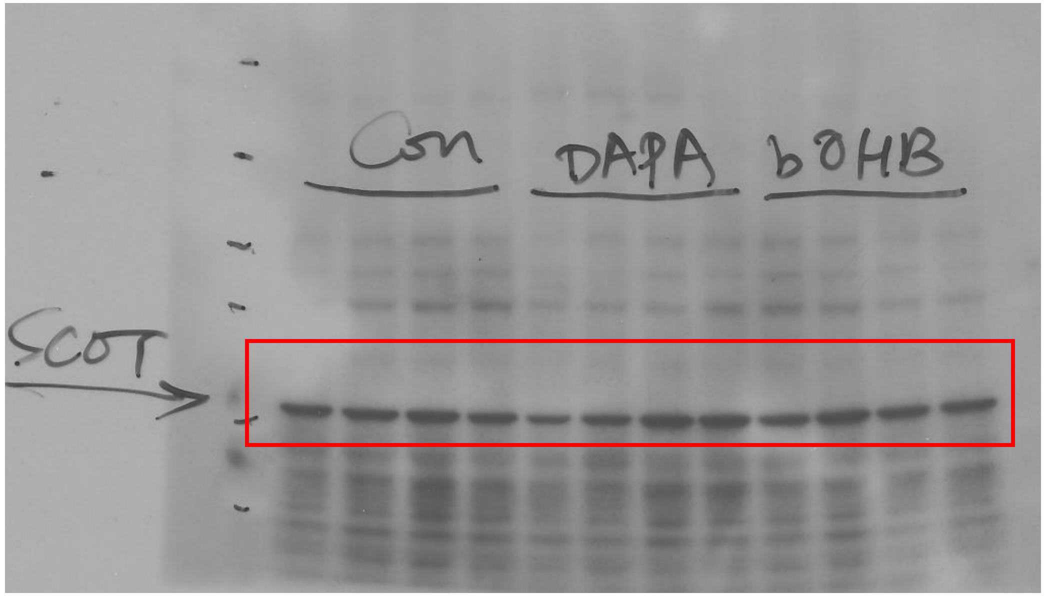

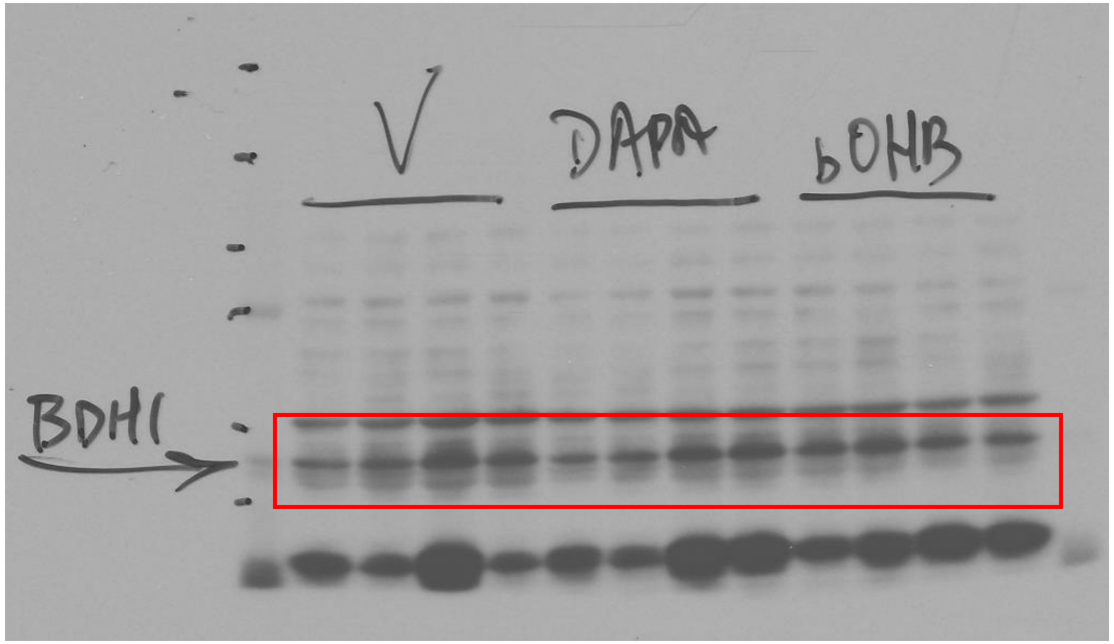

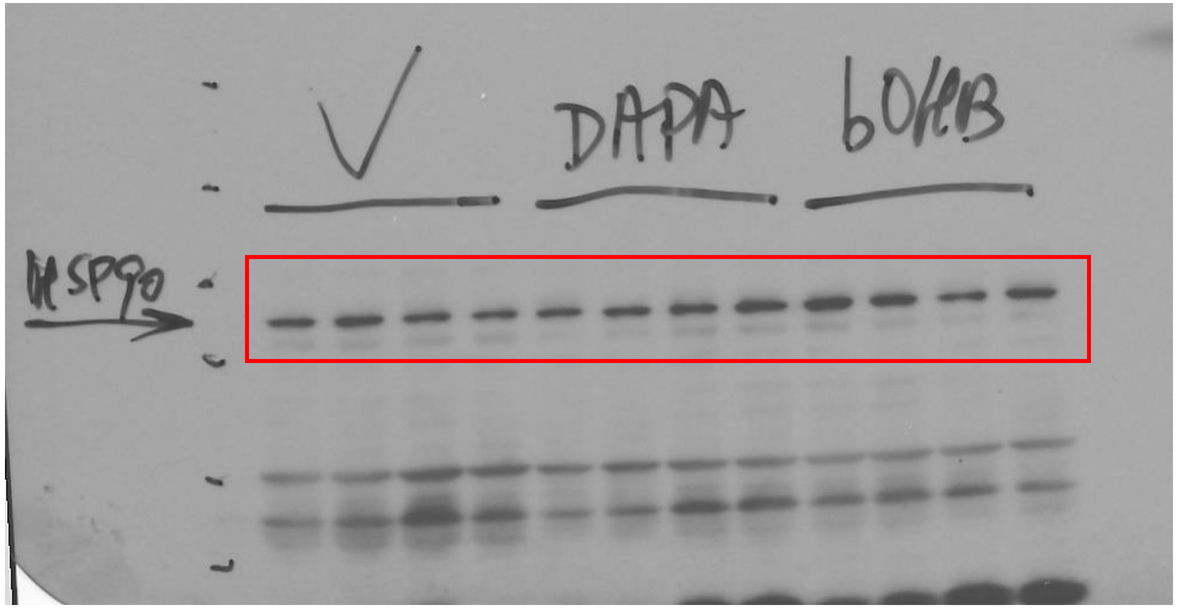

Figure 5G

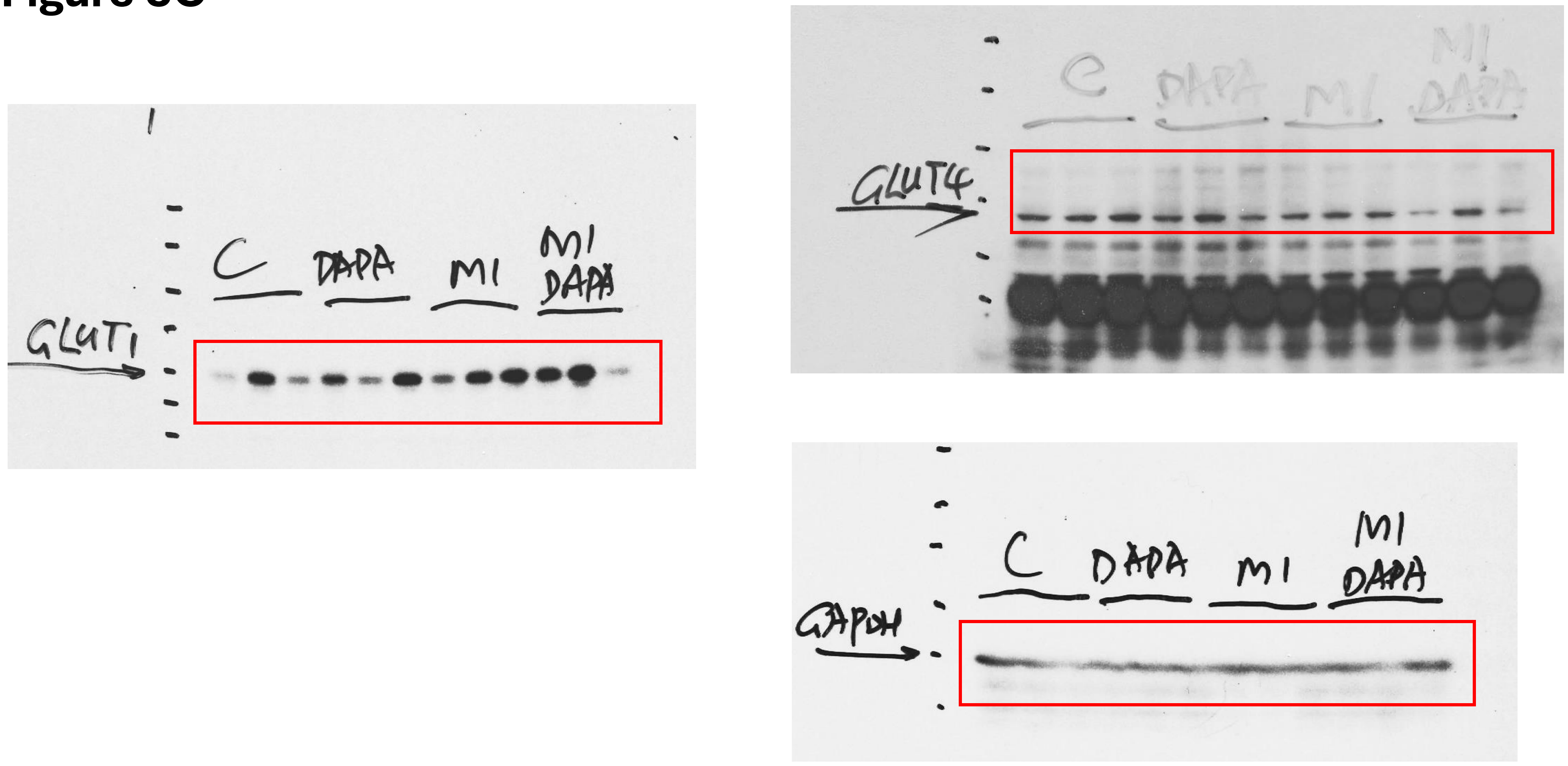
GLUT1
C
DAPA
MI
MI DAPA
GLUT4
C
DAPA
MI
MI DAPA
GAPDH
C
DAPA
MI
MI DAPA

Figure 5H

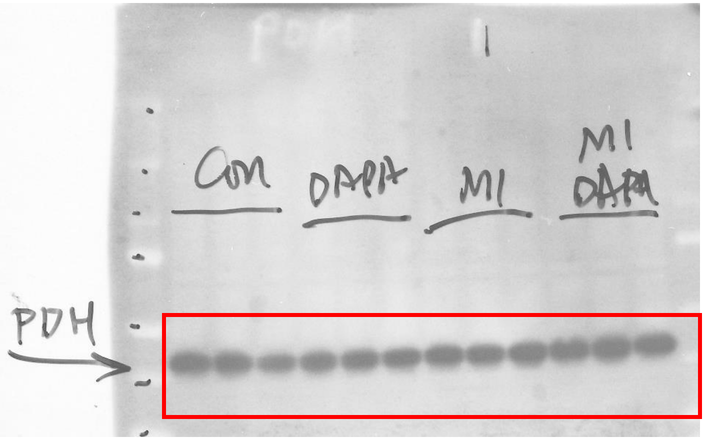
Con
DAPA
MI
MI DAPA
PDH

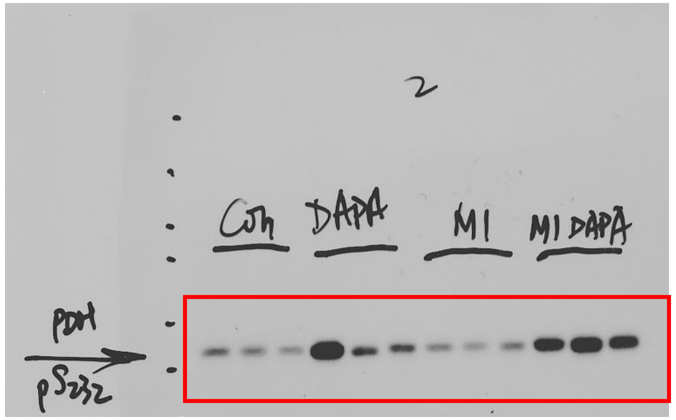
Con
DAPA
MI
MI DAPA
PDH
pS232

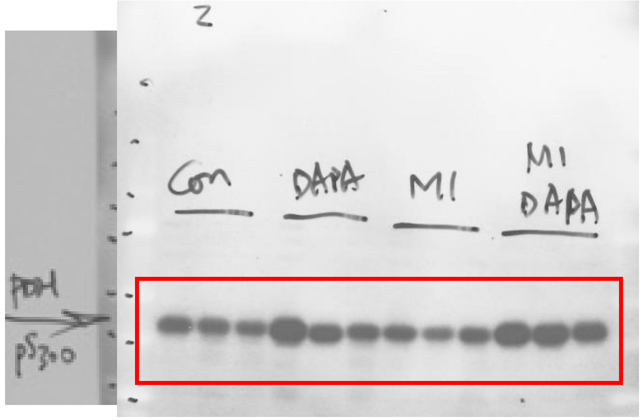
Con
DAPA
MI
MI DAPA
PDH

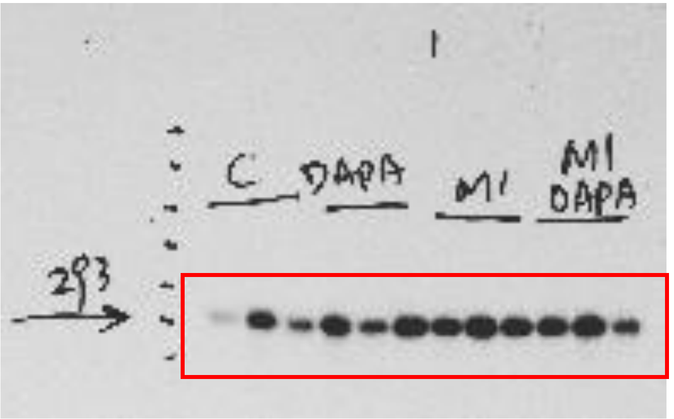
C
DAPA
MI
MI DAPA
293

Figure 5I

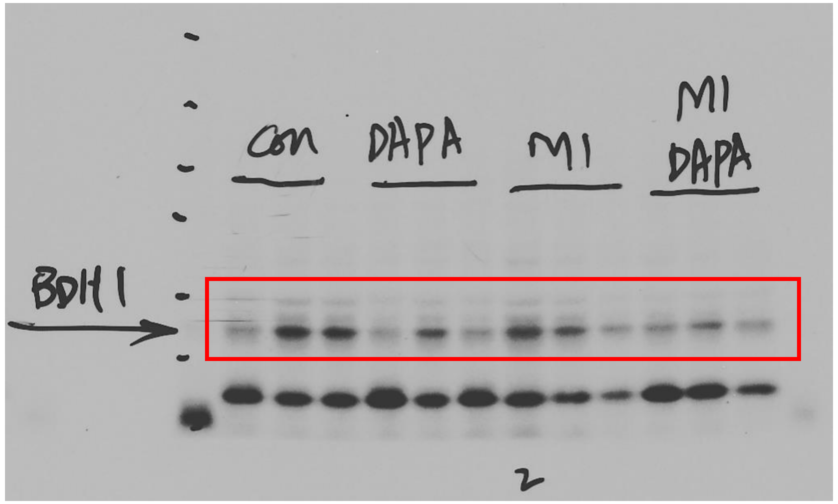

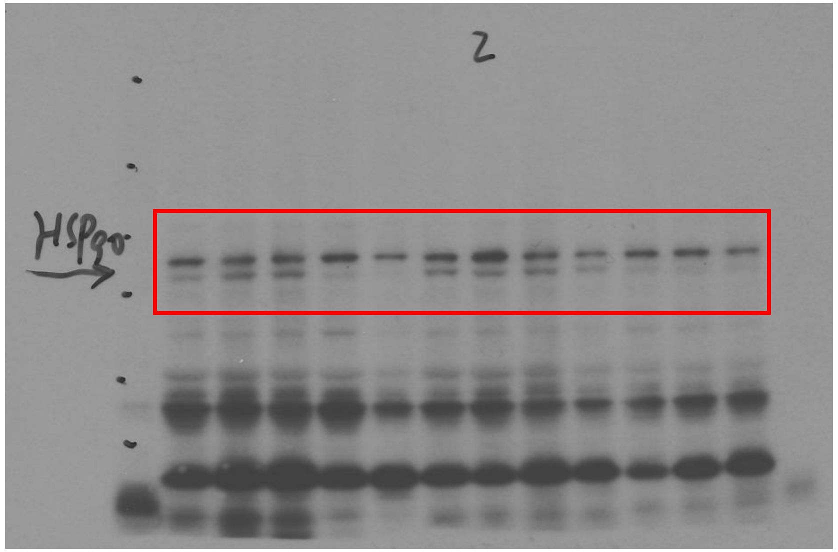

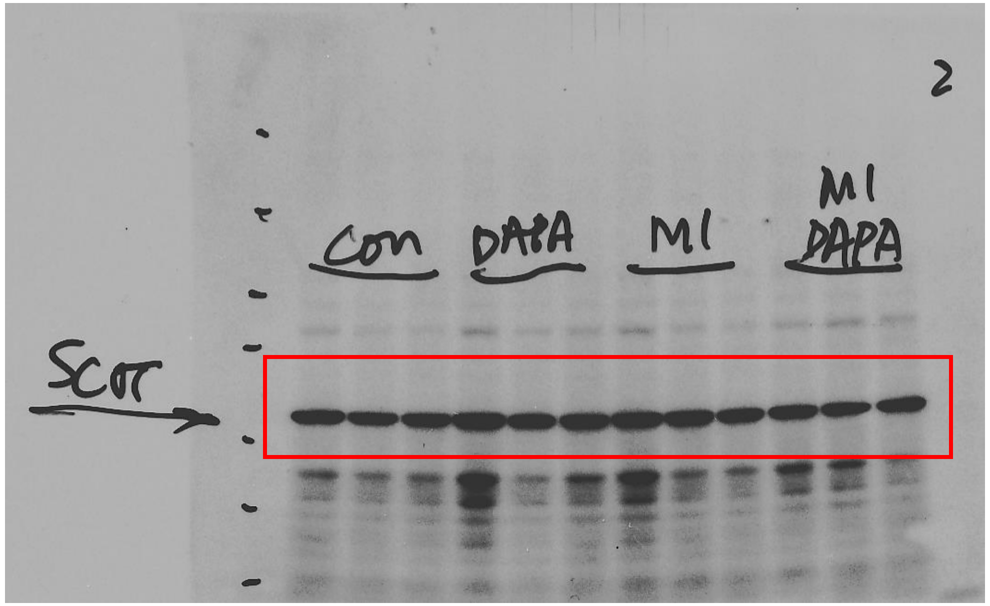

Figure 8E

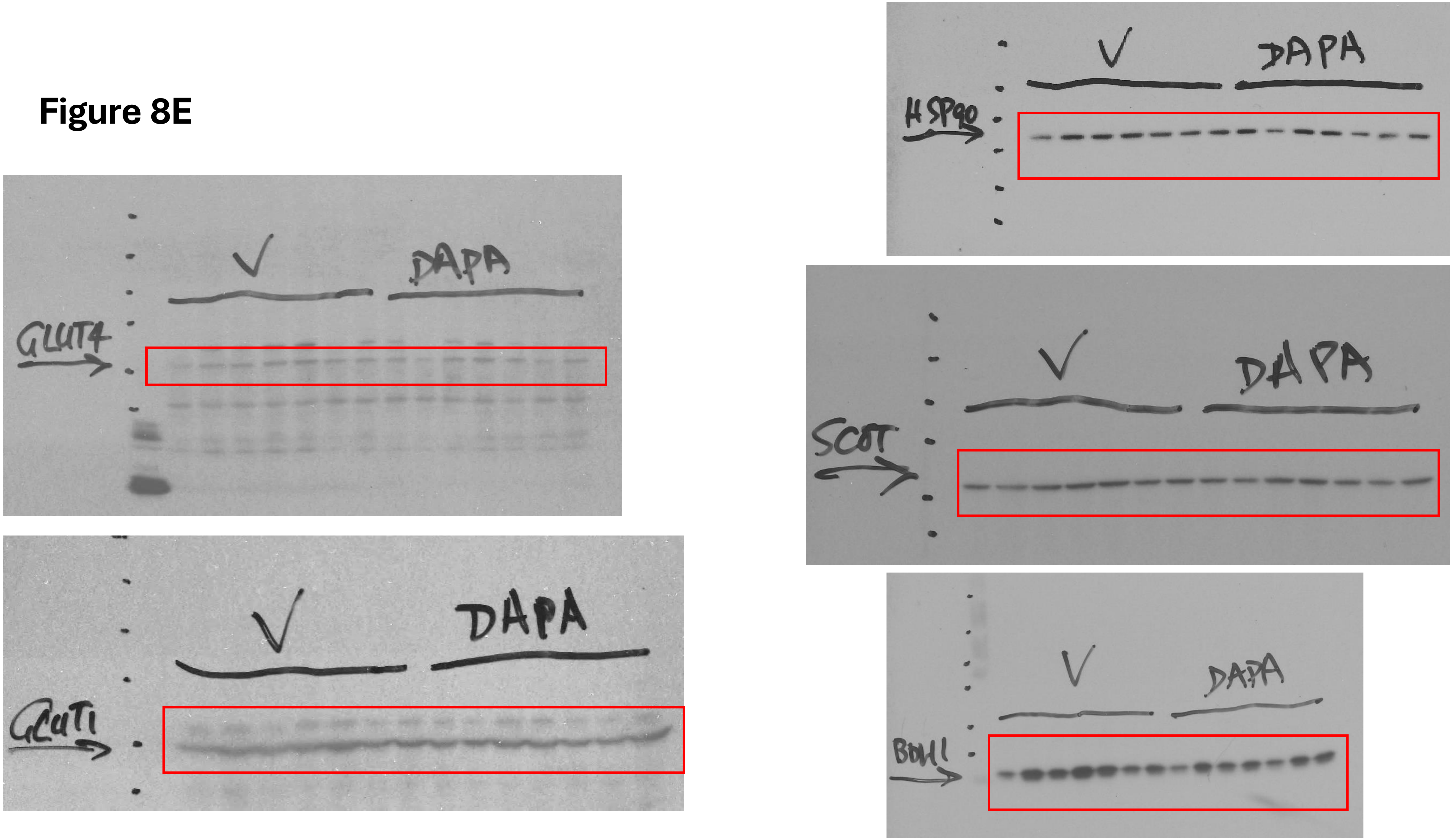

Figure 8F

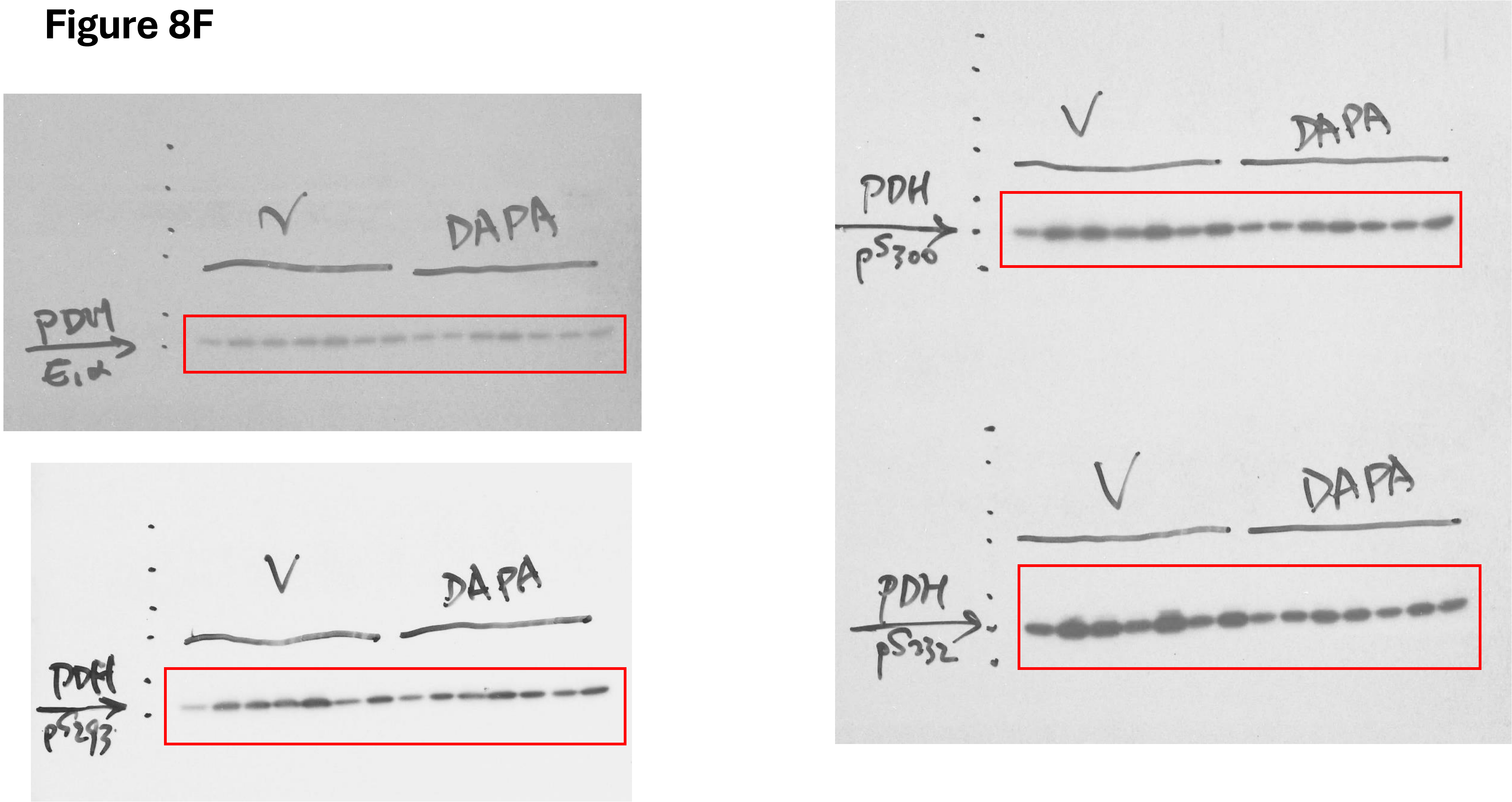
V
DAPA
PDH
E1α
V
DAPA
PDH
pS293
V
DAPA
PDH
pS300
V
DAPA
PDH
pS332
